# Functional reserve mitigates cognitive-motor dual-task costs in older adults: insights from age, cohort, and behavioural strategies

**DOI:** 10.1186/s11556-026-00423-z

**Published:** 2026-06-25

**Authors:** Tian Zhou, Elisa Straub, Andrea Kiesel, Dominic Gehring, Urs Granacher, Aaron Haslbauer, Reto W. Kressig, Roland Rössler

**Affiliations:** 1 Department of Psychology, University of Freiburg, Freiburg, Germany; 2 Department of Sport and Sport Science, Exercise and Human Movement Science, University of Freiburg, Freiburg, Germany; 3 University Department of Geriatric Medicine Felix Platter, Basel, Switzerland; 4 Department of Clinical Research, University of Basel, Basel, Switzerland; 5 Department of Sport, Exercise and Health, University of Basel, Basel, Switzerland

**Keywords:** Cognitive-motor dual-task costs, Functional reserve, Gait speed reserve, Retrospective cross-sectional aging study, Fall risk modelling

## Abstract

**Background:**

Age-related declines in somatosensory and cognitive function impair gait and increase fall risk, particularly under dual-tasking where cognitive and motor demands compete for limited resources. Functional reserve, the difference between one’s maximum capacity and the minimum required for daily functioning, may buffer cognitive-motor interference. This study examines dual-task costs (DTCs) in healthy older adults, focusing on the roles of functional reserve, age-cohort effects, fall history and behavioural strategies such as “Stops Walking When Talking” (SWWT).

**Methods:**

A sample of 4,443 healthy older adults (mean age: 74.8 years, range: 60.05–97.43) completed the Basel Motor-Cognition Dual-Task Paradigm. Participants walked at preferred speed (single task) and performed concurrent cognitive tasks during dual-task walking, targeting semantic (animal naming) and working memory (counting backwards) during walking or as single-tasks. Functional reserve was calculated as the proportional difference between preferred and fast walking speeds. Cognitive and motor DTCs were defined as proportional performance decline from single- to dual-task conditions. Linear mixed models identified predictors of DTCs and assessed logistic-linked fall history. Cohort was residualized from age.

**Results:**

Significant cognitive-motor DTCs emerged, with both semantic and working memory tasks reflecting substantial performance declines when simultaneously walking. Working memory task induced less interference on walking whereas walking interferes more on working memory task, than semantic task, indicating domain-specific competition. Higher functional reserve predicted lower DTCs (motor, *p* < .001; cognitive, *p* = .006), suggesting its protective role. Age and cohort independently influenced DTCs, with more recent cohorts exhibiting higher cognitive-motor DTCs despite comparable ages. Fear of falling strongly predicted fall history (*p* < .001). SWWT was rare (2.8%), yet associated with higher motor DTCs but not falls, suggesting compensatory strategy. Subject-specific cognitive DTC did not correlate negatively to motor DTC after accounting fix effects, thus no trade-off was detected (*r* = − .024, *p* = .270).

**Conclusion:**

Functional reserve mitigates cognitive-motor interference in aging, while SWWT may reflect adaptive compensation. These findings identify functional reserve as potential intervention target and highlight the complexity of resource allocation, given the absence of a motor-cognitive trade-off and the presence of domain-specific effects. Future longitudinal studies should examine behaviour markers and refine cognitive-motor metrics.

**Supplementary Information:**

The online version contains supplementary material available at 10.1186/s11556-026-00423-z.

## Background

Static and dynamic postural control deteriorate greatly with aging [1, 2], and older adults are at greater risk of falls and related injuries [3]. Gait speed, often referred to as the “sixth vital sign” due to its high predictive value for morbidity and mortality [2, 4], is considered “almost the perfect measure” of motor function in aging population [4].

Age-related declines in the somatosensory system and cognitive functions contribute to reduced gait speed. These declines increased mobility limitations, elevated fall risk and sometimes result in a vicious circle of fear of falling and actual falls [5, 6]. For example, the general slowing theory [7] has been proposed to explain the decreased performance in cognitive and motor tasks by assuming a general slowing of executive functions [7, 8] and controlled information processing [9, 10]. Age-related performance declines become more pronounced when individuals perform cognitive and motor tasks simultaneously (i.e., dual-tasking), leading to a significant drop in performance [11–13]. Hereby, the age-related decline in cognitive and sensorimotor performance in dual-task settings seems additive and greater than predicted from general slowing [14–16], indicating that aging exacerbates the preexisting difficulties in dual tasking [17].

**Fig. 1:**
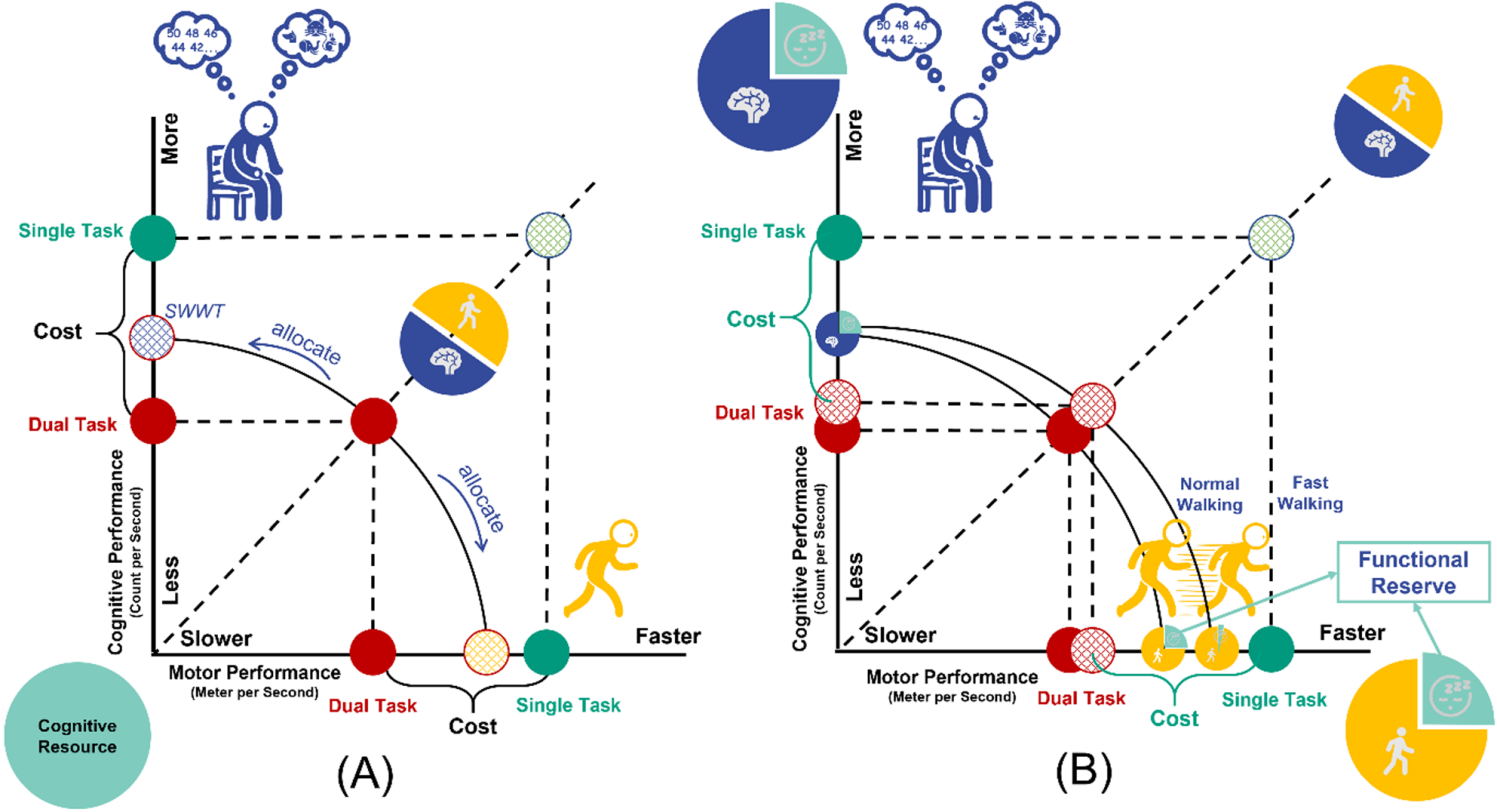
Resource-sharing model [18, 19, 33–37]. **A** Performance Operating Characteristics framework of cognitive-motor interference and resource allocation in healthy older adults. Limited cognitive resources must support both cognitive (in blue) and motor (in yellow) domains during dual-tasking. Concurrence imposes dual-task cost (DTC), the fractional deterioration from single-task. The diagonal line depicts equal sharing; curved paths illustrate biased allocation (e.g., posture-first strategy toward motor). At the limit, edge case “stops walking when talking (SWWT)” reflects a strategic pause of gait to manage interference. **B** The role of functional reserve in cognitive motor interference. Functional reserve (in green) is the difference between an individual’s max- and the regular capacity required, implies spare capacity available for additional task. Baseline demand modulates amount of spare capacity therefore normal walking at self-selected speed leaves more reserve than fast walking. Higher functional reserve, reflected by more increment from normal to fast walking speeds, allows individuals to better maintain performance under dual-tasking

The performance decrement induced by dual-tasking reflects the cost of concurrence (see Fig. 1A), indicating that limited cognitive resource must be shared between the simultaneously performed cognitive and motor tasks [18–20]. This sharing of resource can be biased under prioritization of one task, specifically older adults utilize the “postural first strategy [21]” to prioritize postural control when dual-tasking [22, 23]. In extreme cases, this prioritization manifests as “Stops Walking When Talking” (SWWT), ensuring postural safety by pausing gait and results in the momentary prioritization of the cognitive task [24, 25]. While SWWT has been proposed as a compensatory strategy to reduce cognitive load [26], its relationship with fall history remains debated [24]. Some studies suggest SWWT reflects heightened fall risk [25], while others argue it may be an adaptive response to manage interference [27]. Clarifying this distinction is critical, as SWWT’s low prevalence in healthy older adults (e.g., 2.8% in our sample) limits its utility as a standalone clinical marker. The magnitude of cognitive-motor interference varies by task type, with semantic tasks (e.g., animal naming) typically imposing greater interference to postural control than working memory tasks (e.g., counting backwards) due to higher executive demands [28–32]. However, large-scale empirical comparisons of these task types remain scarce, leaving domain-specific interference patterns unresolved in healthy older adults.

Peel and colleagues [33] found that along the recovery timeline from a fall incident, not only the preferred and fastest possible walking speed of older adults were increasing, the difference between these speeds also significantly increased. Gait speed reserve, coded as the fractional difference between fastest possible and habitual gait speed, captures an individual’s preserved capacity to meet increased task demands and may contribute to reduced fall risk [34, 38]. We conceptualize this measure as an operationalization of functional reserve, that is the difference between an individual’s maximum- and the regular capacity required for daily functioning [34–36], highlighting both the resources available to an individual and the capacity to flexibly allocate them across motor and cognitive tasks.

As illustrated in Fig. 1B, we hypothesize that the functional reserve from the motor domain serves as a resource that can be shared between the walking and the cognitive task in dual task requirements [36]. This functional reserve metric aligns with the Performance Operating Characteristics framework [37], where reserve capacity determines an individual’s ability to meet increased task demands. For example, older adults with higher functional reserve may better sustain gait speed under cognitive load, reducing motor DTCs. While functional reserve has been linked to resilience in aging [35, 36], its role in buffering DTCs remains underexplored. A major aim of our study is to assess whether functional reserve predicts lower DTCs, independent of age or cohort effects.

Building on the Basel Motor-Cognition Dual Task Paradigm [39, 40], our study leveraged a large dataset from Basel Mobility Center (BMC) to address critical gaps in understanding cognitive-motor interference in aging. While prior work has established DTCs as a marker of age-related decline, key questions remain unresolved: the role of functional reserve as a buffer against DTCs, the prevalence and adaptive role of SWWT in healthy older adults, and the independent contributions of cohort effects, where generational differences in cognitive-motor resources may confound chronological aging [41]. Additionally, large-scale comparisons of semantic vs. working memory tasks are rare, limiting conclusions about domain-specific interference. By quantifying DTCs across 4,443 healthy older adults, we aim to disentangle these effects and clarify their mechanistic roles in aging.

## Methods

### Sample

#### Data collection and ethics

All data were collected between 2007 and 2024 at the BMC and the Memory Clinic [42] at the University Department of Geriatric Medicine Felix Platter as part of routine clinical assessments or research studies. These assessments and medical records were conducted collaboratively to aid in diagnosis. The dataset was pseudonymized by removing all personally identifiable information (e.g., name, date of birth, address), with each individual assigned a Unique Patient Number. Only participants who provided consent to share data for research purposes were included. This project received approval from the local Ethics Committee (Ethikkommission Nordwest- und Zentralschweiz, EKNZ, Project-ID 2024 − 01296).

#### Cognitive assessment criteria

Cognitive health was assessed using the Mini-Mental State Examination (MMSE [43]: Score ≥ 24/30 points). In addition, for some participants the Montreal Cognitive Assessment (MoCA [44]: Score ≥ 18/30 points) was accessed. Further, only data of participants with normal cognition as determined by the Memory Clinic [42, 45] were included.

#### Participant selection

We focused on older adults, defined according to the World Health Organization as individuals aged 60 years and above [46]. For the inclusion and exclusion of study participants, study-specific criteria applied, please see Supplemental Material S1 for details, we applied more stringent restrictions to the pooled sample. Participants were excluded if they were younger than 60 or had severe neurological, physical, or psychological impairments according to the cognitive assessment criteria. Only participants who completed the walking tasks without walking aids and who performed all tasks following the Standard Operating Procedure were included in analyses (see Supplemental Material S2 and Figure S1 - S2).

#### Sample characteristics

Among the participants from the year 2007 to 2024, *n* = 4443 (51.3% were female), aged between 60.05 and 97.43 years (Mean = 74.8 years, SD = 7.1), were included for data analysis (see Table 1).

**Table 1:** Participant characteristics

Characteristic	All (*N* = 4443)
Age (years), mean ± SD	74.8 ± 7.1, (*n* = 4443), range [60.0, 97.4]
Cohort (years), mean ± SD	1939.6 ± 8.7 (*n* = 4443), range [1911.6, 1963.4]
Education (years), mean ± SD	12.6 ± 3.7 (*n* = 3942), range [0.0, 35.0]
MMSE, mean ± SD	27.84 ± 1.67 (*n* = 4392)
MoCA, mean ± SD	22.48 ± 3.74 (*n* = 467)
Gender, n (%)	
Male	2163 (48.7)
Female	2280 (51.3)
Recruitment Source, n (%)	
Memory Clinic Routine	2995 (67.4)
Study Purpose (see also Table S1)	1351 (30.4)
Referral for Mobility Diagnosis	93 (2.1)
Missing	4 (0.1)
Previous falls, n (%)	
Yes	1951 (43.9)
No	2358 (53.1)
Missing	134 (3.0)
Fear of falling, n (%)	
Yes	493 (11.1)
No	3784 (85.2)
Missing	166 (3.7)
Stops walking when talking, n (%)	
Yes	123 (2.8)
No	3759 (84.6)
Missing	561 (12.6)
Multiple visits, n (%)	
Yes	1816 (40.9)
No	2627 (59.1)

Values are n (%) unless otherwise indicated. Continuous variables are mean ± SD based on available data (n per row). Previous falls, Fear of falling and stops walking when talking were recorded as binary data (yes/no) based on consistent verbal questioning. Percentages use full sample N. Participants were pooled from multiple studies with varying criteria; generally, inclusion required a minimum age for older cohorts, the ability to walk independently, and without severe cognitive impairment while excluding those with terminal illnesses or severe psychiatric disorders that precluded task compliance, please see also Supplemental Material S1. MMSE = Mini-Mental State Examination, 30 points; MoCA = Montreal Cognitive Assessment, 30 points; Cohort = Birth Year

### Testing procedure

#### Pre-assessment documentation

Participants provided information about fall history, fear of falling, and use of walking aids. The SWWT test was assessed as an ecological measure during participant transfer from the reception area to the gait laboratory. A BMC employee observed whether participants spontaneously halted their gait when engaged in conversation while being accompanied to the testing facility. The administrator’s observation was recorded as binary (yes/no) data. Demographic information and medical profiles were imported from the hospital system.

#### Gait assessment equipment and protocol

Spatiotemporal gait parameters were measured using the GAITRite electronic walkway system (Platinum, CIR Systems, Franklin, NJ, USA) – a 10-meter electronic walkway with integrated pressure sensors. Following an established protocol [40], participants wore their normal clothing and shoes.

To ensure steady-state walking conditions, participants initiated and terminated each walk 2 m before and after the 10-meter measurement walkway, eliminating acceleration and deceleration bias. A test administrator accompanied participants alongside the walkway for safety.

#### Testing trials

Participants completed six testing trials in a fixed order, with rest breaks provided as needed. The administrator verbally explained each task and demonstrated it, if necessary, but no practice trials were allowed. According to the standard operating procedure (see Supplemental Material S2) the testing began with two walking conditions: (1) normal walking, defined as walking along the GAITRite walkway at a self-selected speed, and (2) fast walking, defined as walking as quickly as possible without running or jumping.

Next, participants performed two dual-task walking trials, during which they were instructed not to prioritize one task over the other: 3) working memory dual-task, involving normal walking while counting backward aloud from 50 in steps of two, and 4) semantic memory dual-task, involving normal walking while naming animals aloud from long term memory.

Finally, participants completed two single cognitive tasks while seated: 5) working memory task (counting backward from 50 in steps of two aloud) and 6) semantic memory task (enumerating animals names aloud from long term memory). The duration of the single cognitive tasks was matched to the walking time recorded during the corresponding dual-task trials.

### Variables and indices

Participants’ performance was measured in two domains: motor (walking speed) and cognitive (verbal transcript). Walking speed was calculated as the distance between the first and last footstep, divided by ambulation time, representing mean normalized speed. The verbal transcript included correct numbers or animal names, along with hesitations, repetitions, self-corrections, and expressions; mistakes were rare and excluded from the analysis [39].

To control for the length of walking times that determine the cognitive task time in dual-task and single-task conditions, we coded the number of counted numbers or correct animal names as “occurrences per second” by dividing the number of occurrences by ambulation time. For example, the number of animal names during the semantic memory task while walking is denoted as *n*_*semantic*&*walking*_, and the time spent as *t*_*semantic*_, with performance normalized as occurrences per second:

$$\:{N}_{semantic\&walking}=\frac{{n}_{semantic\&walking}}{{t}_{semantic}}$$

DTC refers to the performance decrement in dual-task versus single-task situations and is typically positive, indicating a loss from dual-tasking. Negative values rather indicate a dual-task gain [47]. For example, the motor DTC on the semantic task, representing the performance drop with the extra motor task, is computed as $\:{N}_{semantic}-{N}_{semantic\&walking}$.

Dual-task performance is sensitive to individual differences, requiring normalization at the individual level [47]. DTC for older adults is commonly expressed as a percentage of single-task performance:

$$\:Cognitive\_DT{C}_{semantic<-walking}=\frac{{N}_{semantic}-{N}_{semantic\&walking}}{{N}_{semantic}}\mathrm{*}100\%$$

Here, “cognitive” refers to the measurement domain, “semantic” to the cognitive task, and “semantic <- walking” to the change in semantic task performance under walking interference. The DTC for the working memory task is computed similarly, with “N” as the number correctly counted per second.

For motor DTCs, if *V*_*walking*_ is normal walking speed and *V*_*walking*&*semantic*_ is walking speed while naming animals, the motor DTC is:

$$\:Motor\_DT{C}_{walking<-semantic}=\frac{{V}_{walking}-{V}_{walking\&semantic}}{{V}_{walking}}\mathrm{*}100\%$$

“Motor” refers to the measurement domain, “semantic” to the cognitive task, and “walking <- semantic” to the change in walking performance under semantic memory task interference. The DTC for the working memory task is computed similarly, with “walking <- working” indicating the drop in motor task performance due to the working memory task.

The difference between usual and maximum speed represents one’s functional reserve in the motor domain, coded as the fractional difference between walking speeds in single-task conditions:

$$\:Functional\_Reserve=\frac{{V}_{fast}-{V}_{walking}}{{V}_{fast}}\mathrm{*}100\%$$

Age was residualized against the year of birth to mitigate age-cohort collinearity. We modelled residualized cohort alongside standardized age to predict DTCs and fall history, a well-supported strategy to control for cohort effects in multivariate analyses [48–50].

Fear of falling, SWWT, and fall history were recorded as binary data via consistent verbal questioning. Qualitative elaborations provided by participants were not included in the analysis. A binomial link function was applied to fall history to predict the likelihood of prior falls, providing ecological validity for future fall risk.

### Statistical analysis

Data preprocessing was conducted in Python 3.12.0 [51], and statistical analyses in R 4.5.1 [52]. Continuous variables were examined for distribution using histograms, skewness, kurtosis, and Q-Q plots. Motor and cognitive DTCs (semantic and working memory) were log-transformed to accommodate non-normal distributions.

Normality and multicollinearity diagnostics preceded modelling. Histogram and Q–Q plots revealed non-normal distributions across all DTC outcomes: cognitive costs seemed multimodal with large deviation, and motor cost was strictly positive and right-skewed (see Fig. 3A). Shapiro–Wilk tests confirmed non-normality (all *p* < .001). Applying log-transformation to cognitive and motor costs improved symmetry. Pearson correlations and variance inflation factors (VIFs) indicated no multicollinearity (|r| < 0.7, VIFs < 1.05).

Missing data arose mainly from incomplete cognitive responses or missing fall history, fear of falling, and SWWT values. Such missingness is typical in large datasets, consequently sample sizes varied by analysis.

Paired-sample *t*-tests compared single- and dual-task performance to validate DTCs, the relative performance drops between conditions. Cohen’s d and power were computed using the *pwr* package [53].

Generalized linear mixed-effects models (GLMMs) assessed predictors of cognitive and motor DTCs respectively. Fixed effects included residualized age, task type (semantic vs. working memory), functional reserve, fear of falling, SWWT, and fall history. Participant ID was a random intercept. Overdispersion was handled by excluding random intercepts when needed. Model selection followed AIC/BIC and residual diagnostics. All assumptions, transformations, and model selections were tested for robustness.

To examine evidence for a motor–cognitive trade-off, we estimated the association between cognitive and motor dual-task interference after accounting for model predictors. Specifically, we computed the Pearson correlation between standardized residuals derived from the respective linear mixed models [54, 55]. This approach captures the shared unexplained variance between domains, analogous to a partial correlation, and can be interpreted as residual coupling between cognitive and motor performance beyond modelled effects [55]. This approach approximates the residual covariance between outcomes that would otherwise be estimated in a multivariate multilevel model framework.

GLMMs with a binomial logit link predicted fall history from DTCs, task type, age, functional reserve, SWWT, and fear of falling. Participant ID was modelled as a random intercept; interaction terms were tested and removed if nonsignificant. The random intercept was omitted when overdispersion was present to improve model stability.

A two-tailed significance threshold of α = 0.05 was applied, with model coefficients reported alongside 95% confidence intervals and standardized effect sizes. To account for multiple comparisons, we used a Bonferroni correction when applies (α = 0.025).

## Results

### Emergence of cognitive-motor DTC

**Fig. 2:**
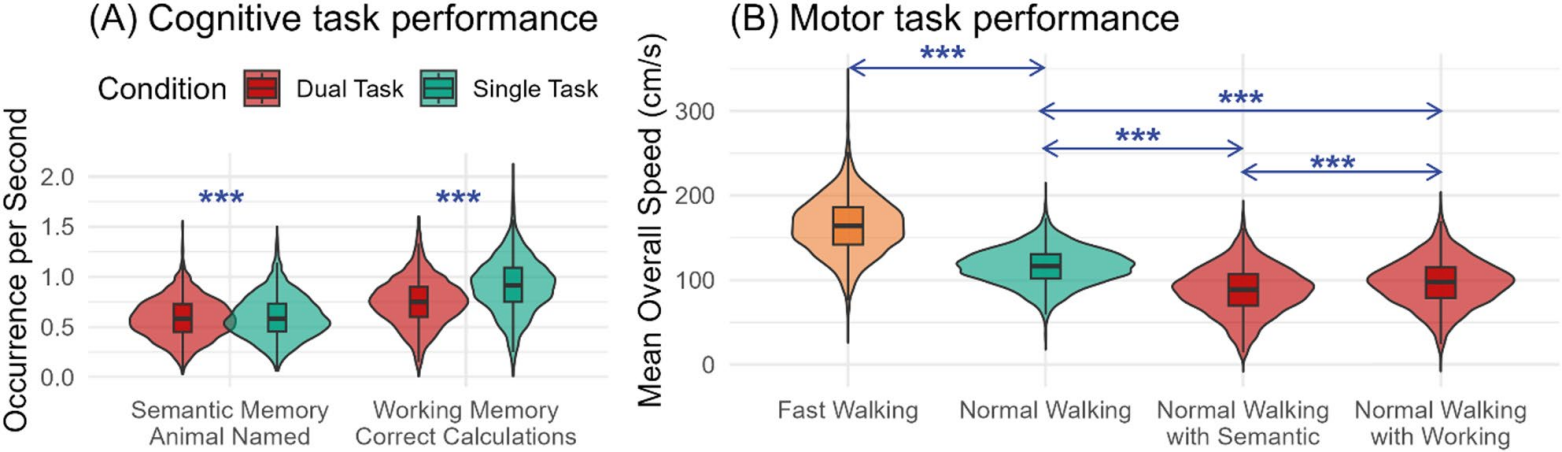
Violin plots of cognitive (**A**) and motor (**B**) performance in single and dual task conditions across semantic and working memory tasks. Both semantic and working memory tasks showed significant performance reductions under dual-tasking (the statistical significance was likely due to the large sample size), with walking speed also significantly declined during cognitive tasks, denoted by asterisks (see Table S2 for descriptive statistics)

To assess the impact of dual-tasking on performance, we compared correct responses (animals or numbers per second) and walking speed between single- and dual-task conditions across semantic memory, working memory, and gait. As shown in Fig. 2A, both cognitive tasks exhibited significant DTCs. In the semantic memory task, performance while walking (M = 0.584, SD = 0.201) was significantly lower than while sitting (M = 0.599, SD = 0.214), *t*(1638) = -4.25, *p* < .001, with a mean difference of -0.016 and 95% CI [-0.023, -0.008]. Similarly, working memory performance declined under dual-tasking (walking: M = 0.747, SD = 0.239; sitting: M = 0.908, SD = 0.276), *t*(1586) = -39.65, *p* < .001, with a mean difference of -0.165 and 95% CI [-0.173, -0.157].

**Fig. 3:**
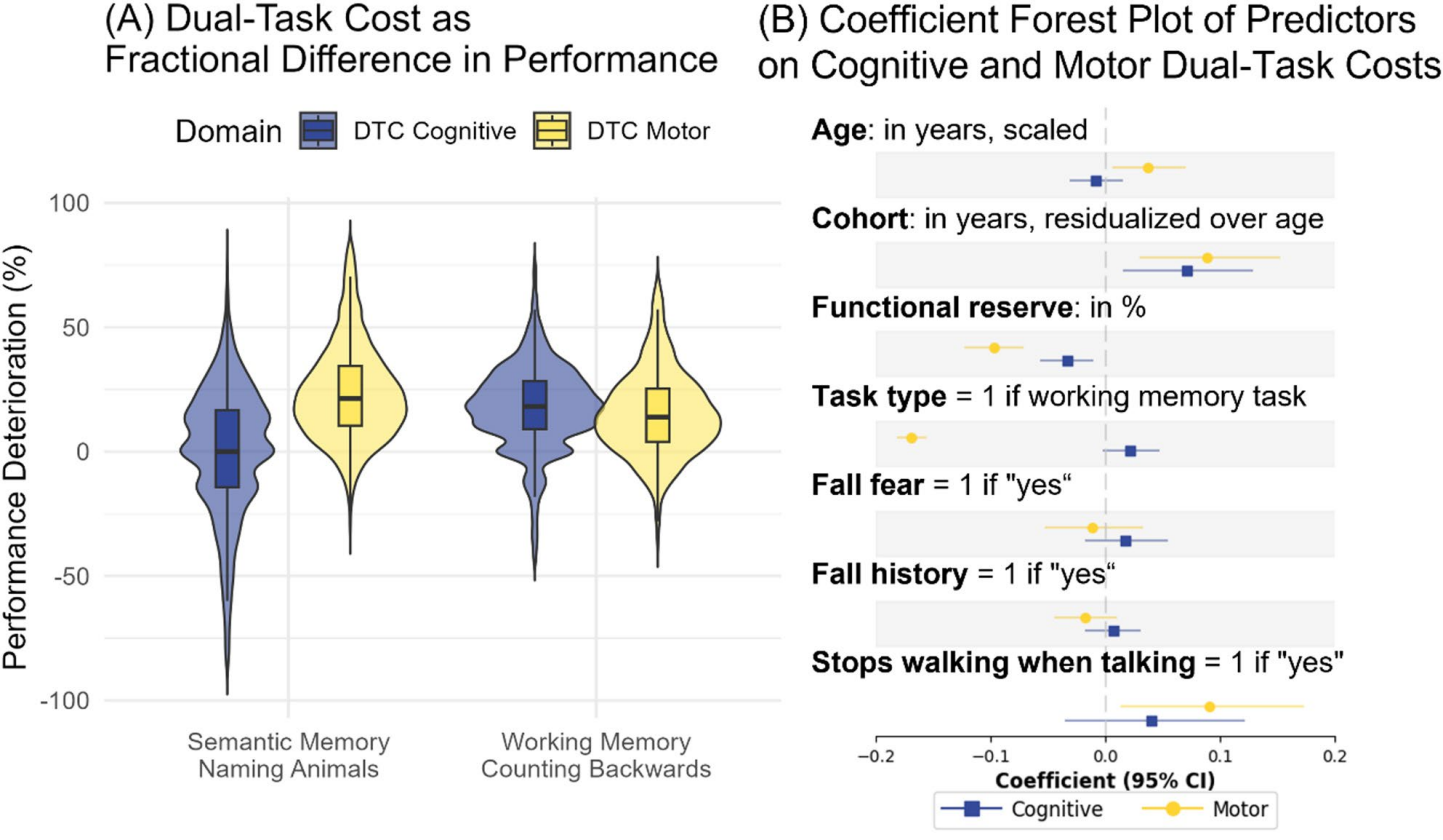
Cognitive and motor dual-task costs (DTCs) across semantic and working memory tasks, with linear mixed model (LMM) predictions. **A** DTCs index fractional difference between single and dual task performance, were robust for motor tasks. Descriptively cognitive costs were task-dependent, simultaneously walking introduced more interference for working memory than semantic. Whereas compared to only walking (motor single task), adding simultaneous semantic memory task introduced more interference on walking than working memory task introduced. These task dependent differences of dual-tasking were confirmed by the models fitted. **B** LMM analysis for motor DTCs (yellow circle) revealed that higher functional reserve significantly reduced motor DTCs. Age and cohort were significant predictors, with older individuals and more recent cohorts exhibiting higher motor DTCs. For cognitive DTCs (blue square), functional reserve showed a significant negative association. More recent cohort significantly predicted higher cognitive DTCs. However, age was not predictive. Fear of falling and fall history did not affect either DTCs. SWWT was significantly associated with motor DTCs but had no significant effect on cognitive DTCs. Please see detailed model diagnosis in Supplementary Materials S4-S7

As depicted in Fig. 2B, walking speed also decreased significantly when performed concurrently with cognitive tasks. Compared to normal walking (M = 115.94 cm/s, SD = 21.86), walking speeds were lower during the semantic memory (M = 87.69 cm/s, SD = 28.46) and working memory tasks (M = 96.95 cm/s, SD = 27.76. While both cognitive tasks significantly reduced walking speed (Fig. 2B), the magnitude of motor DTCs did not differ between semantic and working memory tasks (Fig. 3A). All paired comparisons between single- and dual-task walking speeds were statistically significant (*p* < .001).

### LMM prediction of cognitive DTC

Predictors of cognitive DTC were analysed using LMMs including task type, functional reserve, standardized age, cohort (residualized birth year), fear of falling, SWWT, and fall history, with random intercepts for individuals.

Cognitive task type did not significantly predict cognitive cost, (*β* = 0.04, *p* = .076). Our data offered evidence towards additional walking task interfere more with working memory task. We also found a significant negative association between functional reserve and cognitive DTCs (*β* = -0.07, *p* = .006). Age was not significantly associated with cognitive DTCs (*β* = -0.02, *p* = .502). Cohort was a significant predictor, *β* = 0.06, *p* = .011, with more recent cohorts experiencing higher cognitive costs.

Model diagnostics indicated good validity (AIC = 7644.7, BIC = 7703.7). Random effects analyses revealed substantial variability between subjects in cognitive costs, with an estimated intercept SD of 0.23 and a larger residual SD of 0.97, indicating notable individual differences in DTCs.

### LMM prediction of motor DTC

We first fitted a fixed-effects linear model to predict log-transformed motor DTC. Significant predictors were task type (*β* = -0.37, *p* < .001), functional reserve (*β* = -0.09, *p* < .001), cohort (*β* = 0.08, *p* = .004), and task X cohort interaction (*β* = 0.09, *p* = .042). Fear of falling, age, SWWT, and fall history were not significant. The fixed-effects model explained 5.1% of variance (*R*² = 0.051), highlighting substantial subject-level variability unaccounted for by fixed predictors alone.

To address this subject-level variability and account for the nested structure of participants with multiple visits, we fitted a LMM with random intercepts for participants. The random intercept was employed to partition the variance between inter-individual baseline differences and the specific effects of the cognitive tasks. The previously significant task X cohort interaction was removed from the final model due to minimal improvement in AIC/BIC and high multicollinearity with main effects (VIF > 2.0). This model significantly improved fit over the fixed-effects model (χ²(1) = 788.12, *p* < .001), increasing the conditional *R*² to 0.607 (vs. marginal *R*² = 0.051). This substantial increase in conditional *R*^2^ indicates that while subject-specific variance is dominant, the LMM effectively isolates the fixed predictors from this baseline noise.

The final LMM retained significant effects of task type (*β* = -0.19, *p* < .001), cohort (*β* = 0.05, *p* = .003), age (*β* = 0.04, *p* = .018) functional reserve (*β* = -0.11, *p* < .001), and SWWT (*β* = 0.03, *p* = .020). That participants with older age, more recent cohort or lower functional reserve experienced higher motor DTC. Their motor DTC was also expected to be higher when performing the semantic memory task or presented SWWT. Fear of falling and fall history were not predictive. Diagnostic plots showed acceptable residual distributions and minimal multicollinearity. Bootstrap intervals supported the robustness of parameter estimates. All reported *p*-values for DTC analyses met Bonferroni correction threshold.

### Correlation of cognitive and motor DTCs

The correlation between standardized residuals (after controlled for fixed effects) from the cognitive and motor DTC models was small and non-significant (*r* = − .024, *p* = .270), indicating no evidence for a motor–cognitive trade-off after accounting for model predictors.

### GLMM prediction on the probability of having a fall history

To predict fall history, we fitted a generalized linear mixed model (GLMM) with a binomial logit link, guided by our theoretical framework that fall risk is influenced by demographic (cohort, age, fear of falling), behavioural (SWWT), and performance-based (combined DTCs, functional reserve) factors. The initial model included all hypothesized predictors as fixed effects. A random intercept for participants (*1|id*) was included to account for individual variability in baseline fall risk.

While the random intercept improved model interpretability, it introduced overdispersion with dispersion ratio = 1.32. To resolve this, we removed the random intercept. After removal, overdispersion declined (*p* < .001), with dispersion ratio improved to 1.069. Additionally, residual autocorrelation decreased (*p* = .004) and VIFs remained low (all VIFs < 1.1), indicating no multicollinearity. Random slope models did not improve model fit, as the random slope for cost was effectively zero.

**Fig. 4:**
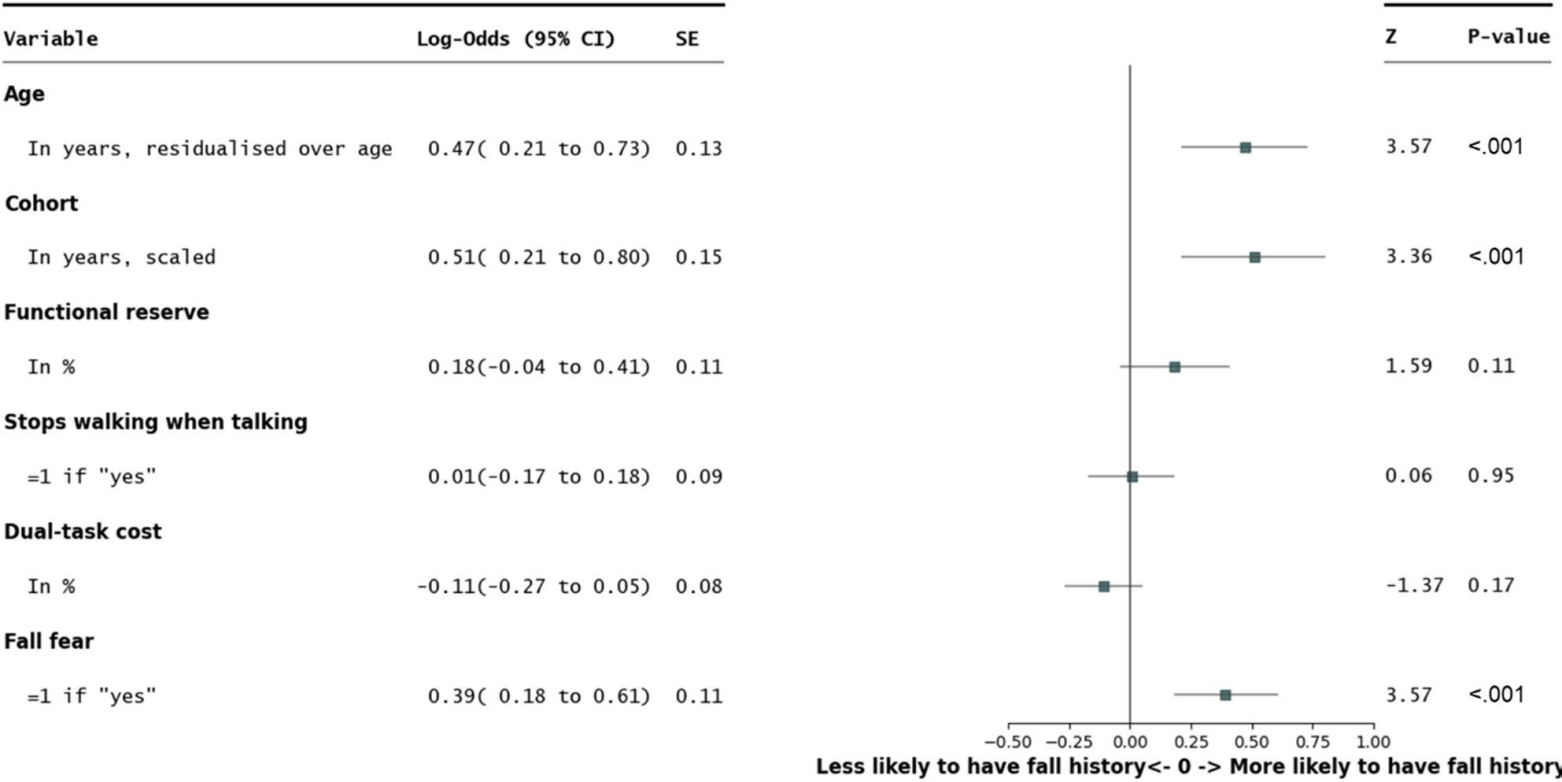
Log-Odd Forest Plot of Predictors on Fall History. The plot visualizes the relative contribution of each predictor to the likelihood of prior falls, with log odds ratio on the x-axis. Error bars represent 95% confidence intervals. A log odd of 0 indicates a 50% probability, positive log odds indicate more likely and negative log odds indicate less likely to have a fall history. Please see detailed model diagnosis in Supplementary Materials S4-S7

The final model retained all predictors of falls regardless of significance to test our theoretical hypotheses. Model fit was optimized (AIC = 13,142.3, BIC = 13,221.4). As shown in Fig. 4, significant predictors were cohort (*β* = 0.51, *p* < .001), age (*β* = 0.47, *p* < .001), and fear of falling (*β* = 0.39, *p* < .001). Individuals from more recent cohorts and older ages were more likely to report falls, and fear of falling remained a strong predictor. In contrast, SWWT (*β* = 0.01, *p* = .951), combined DTC (*β* = -0.11, *p* = .170), and functional reserve (*β* = 0.18, *p* = .110) were nonsignificant. The model’s random intercept structure accounted for variability in fall risk across individuals.

## Discussion

### Summary of findings

Our study replicated the emergence of cognitive–motor DTCs in relatively healthy older adults, demonstrating consistent performance decrements across both cognitive and motor domains under dual-tasking. Descriptively cognitive DTCs were task-dependent, simultaneously walking introduced more interference for working memory than semantic. Whereas compared to only walking (motor single task), adding simultaneous semantic memory task introduced significantly more interference on walking (higher motor DTCs) than walking memory task introduced [28, 32]. Age was a significant predictor of motor DTCs, suggesting that older adults experience greater motor interference under dual-task demands. However, age was not significantly associated with cognitive DTCs, indicating that age-related effects are more pronounced for gait than for cognition.

Cohort effects revealed a counterintuitive trend: participants from more recent cohorts exhibited higher cognitive DTC, Motor DTC and fall risk at equivalent ages, challenging assumptions of uniform generational improvements in mobility [44, 48]. Nevertheless, this finding highlighted the necessity of investigating cohort effect in shaping fall history, independent of chronological aging.

Functional reserve was a protective factor, relating to reduced motor and cognitive DTCs, consistent with its buffering role. Fear of falling robustly predicted higher likelihood of having fall history. By contrast, neither combined DTCs nor functional reserve predicted fall history, highlighting that behavioural and demographic factor are more associated with falls than performance-based dual-task metrics. SWWT (observed in 2.8% of participants) was associated with higher motor DTCs but showed no link to fall history. While its low prevalence limits interpretability, SWWT may reflect adaptive resource reallocation in healthy older adults, though its necessity could also signal underlying decline in dual-task capacity. Future longitudinal work is needed to disentangle these possibilities.

Taken together, these findings reveal both shared and domain-specific influences on dual-task performance in aging. Functional reserve and strategic adaptations (e.g., Postural First Strategy, SWWT) mitigate interference, but real-world fall history appears to track more closely with demographic and behavioural markers than with DTC magnitude alone.

### Age-cohort effect on cognitive-motor interference

Our findings reveal a complex influence of aging and cohort effects on cognitive and motor DTCs, with distinct patterns for motor and cognitive domains. Cognitive DTCs were better captured by birth cohort than chronological age after residualizing cohort on age. Motor DTCs increased with both chronological age and cohort, suggesting additive effects of within-individual decline [14, 41] and between-cohort differences [50, 56]. In contrast, cognitive DTCs were better predicted by cohort than age. This aligns with evidence that cognitive aging trajectories are shaped by generational exposures, such as more prevalent long-sitting [56], but not chronical aging alone.

The positive cohort effect (i.e., higher cognitive and motor DTCs in more recent cohorts) challenges assumptions of uniform generational improvements in mobility. While healthcare advances have extended lifespan, they have also increased multimorbidity survival, which may exacerbate DTCs despite comparable ages [57]. Additionally, reporting biases (e.g., heightened awareness of fall risk) and secular trends (e.g., sedentary lifestyles [56]) could inflate perceived DTCs in younger cohorts [58]. These findings underscore the need to disentangle aging-related decline from cohort-specific vulnerabilities in dual-task research.

### Functional reserve and resource allocation

The general slowing theory [5] posits that aging-related processing speed declines and cautious decision-making contribute to higher DTCs [8, 12]. In our study, participants produced fewer correct numbers or names per second when dual-tasking, reflecting slowed working memory processing and long-term memory retrieval. Our results support a dynamic resource allocation model in which functional reserve buffers cognitive-motor interference [35], but its effects are domain- and task-sensitive.

Higher functional reserve, which operationalized as the proportional difference between preferred and fast walking speeds, was strongly associated with lower motor DTCs (*p* < .001) and reduced cognitive DTCs (*p* = .006). This offers potential support of resource sharing (see Fig. 1B), implying that reserve capacity enables individuals to sustain performance under dual-task demands. For example, older adults with greater functional reserve may prioritize gait stability by reallocating resources from cognitive tasks, thereby reducing motor DTCs.

However, we found no evidence for a fixed motor–cognitive trade-off, as the residual association between cognitive and motor dual-task costs was small and non-significant (*r* = − .024, *p* = .270). This pattern does not support models that assume a rigid competition for shared resources. Instead, it is more consistent with flexible or context-dependent allocation accounts, in which interference may not manifest as a simple inverse relationship between domains [18]. To further characterize patterns of resource allocation, we additionally explored composite indices, including the combined dual-task effect (cDTE) and modified attention allocation index (mAAI) as the functions follow [59]. We incorporated these alternative metrics into our analysis pipeline and by pairing our residual-based trade-off indices with this additional metric, our analysis better captures the same theoretical construct from complementary perspectives and thereby strengthens the interpretation of functional reserve as a buffer within a resource-sharing framework rather than as evidence for a rigid motor-cognitive trade-off. These analyses were exploratory and intended to provide a more descriptive account of how interference may be distributed across domains.

Similarly, we take semantic memory task as an example. Here cognitive (*semantic* count of animal names per second *N*) and motor (*walking* speed *V*) performance declined due to dual-tasking (*walking&semantic*), “*semantic <- walking*” implies the change in semantic task performance under walking interference.

$$\:c{DTE}_{semantic}=\frac{{V}_{walking\&semantic}\:*{\:N}_{walking\&semantic}\:-\:{V}_{walking}\:*\:{N}_{semantic}}{{V}_{walking}\:*\:{N}_{semantic}}*100\%$$

$$\:m{AAI}_{semantic}=Motor\_DTC^\prime\:_{semantic<-walking}\:-\:Cognitive\_DTC^\prime\:_{semantic<-walking}$$

**Fig. 5:**
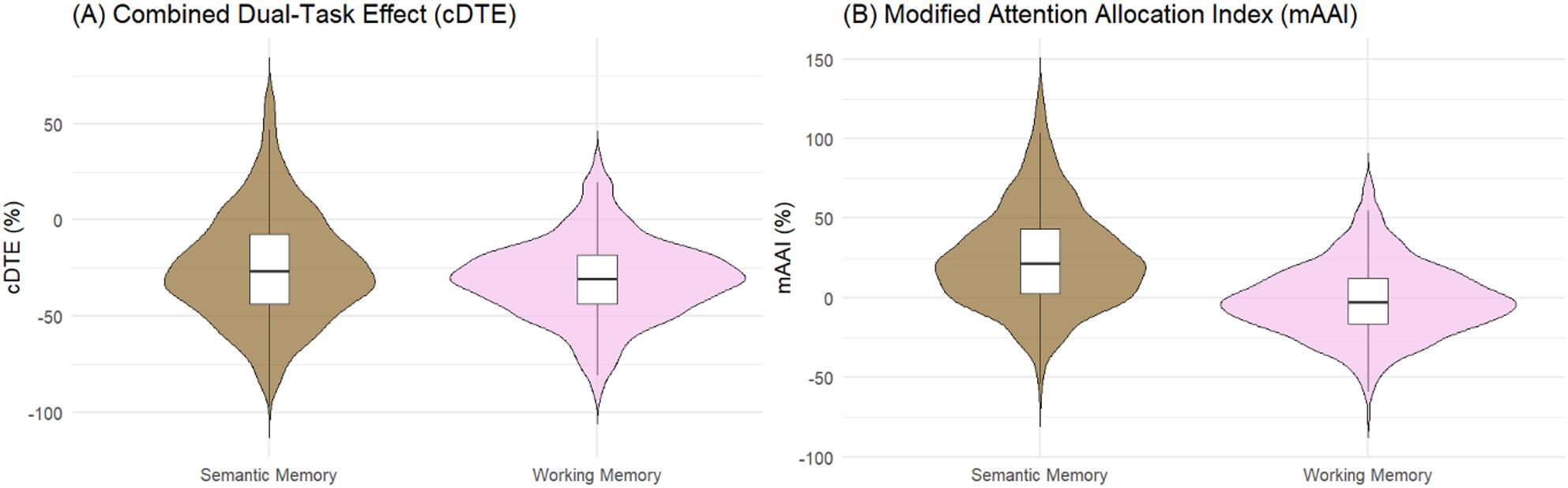
Cognitive-motor dual-task recourse allocation. **A** Violin plot of combined dual-task effect (cDTE). Negative cDTEs reflected interference between cognitive and motor domains. **B** Violin plot of modified attention allocation index (mAAI). The mAAI for semantic memory task was positive, reflected a shift in attention toward the motor task. Working memory task was close to 0 with mAAI, implies no dual-task priority when performing working memory task and walking task simultaneously

While we found no evidence for a fixed motor–cognitive trade-off at the overall level, task-specific patterns suggested a more flexible allocation of resources. In particular, both semantic and working memory tasks implied an interference between cognitive and motor tasks (see Fig. 5A). However, positive mAAI of semantic memory task (M = 26.92, SD = 36.12) implies a shift in attention toward the motor task, whereas mAAI of working memory task was close to 0 (M = -0.01, SD = 26.11) which implies no dual-task priority (see Fig. 5B). This dissociation indicates that dual-task interference may not manifest as a stable inverse relationship between domains, but rather as a context-dependent prioritization strategy. Such flexibility is consistent with adaptive accounts of dual-tasking, in which individuals dynamically allocate resources depending on task demands and perceived risks, rather than operating under a rigid, zero-sum constraint.

### SWWT a compensatory strategy or marker of decline?

The low prevalence of SWWT (2.8%) in our healthy cohort contrasts with clinical populations (e.g., 41% in patient with stroke history [26]), suggesting that SWWT may emerge later in the aging process or under specific conditions. While SWWT was associated with higher motor DTCs, it showed no link to fall history. This might indicate that SWWT may serve as an adaptive strategy in healthy older adults, and reflect proactive resource reallocation to manage interference, especially when dual-task effort exceeds capacity [22]. It may also serve as a decline marker, particularly SWWT may signal reduced dual-task capacity [24–26] in clinical conditions. Longitudinal studies are needed to determine whether SWWT transitions from compensation to pathology with aging or disease progression. For now, its low prevalence and lack of association with falls suggest that SWWT is not a robust clinical marker in healthy older adults, but rather a behavioural indicator of resource limits.

In general, our findings favour a flexible strategy-based resource allocation that prioritizes postural control when risk is salient, rather than a simple cognitive-motor trade-off.

### Limitations and outlook

This study provides valuable insights into cognitive-motor interference in healthy older adults, but several methodological and conceptual limitations warrant consideration. First, the cross-sectional design inherently limits causal inferences. While we observed associations between functional reserve, DTCs, and fall history, longitudinal studies are needed to determine whether these relationships reflect causality or are merely correlations. For instance, the relationship between fear of falling and fall history is known to be bidirectional, evident from both prospective [60] and longitudinal studies [61]. However, does higher functional reserve protect against future falls, or does it simply reflect preserved capacity at a single time point?

A second set of limitations stems from methodological constraints in our assessment protocols. The fixed trial order (e.g., cognitive single-task followed by dual-task) may have introduced artificial confounds, such as practice or fatigue, which could artificially inflate or underestimate DTCs. For instance, in the animal naming task, the initial attempt requires retrieval from long-term memory, whereas the subsequent dual-task attempt may partially rely on short-term memory representations of the previously named animals. Such a carry-over effect could potentially lead to an underestimation of the cognitive DTC. While order effects were consistent across participants thus unlikely to bias group comparisons, they may have obscured the true magnitude of DTCs. Future studies interesting in estimating the size of DTCs should counterbalance task order to isolate cognitive-motor interference.

The operationalization of SWWT also presents challenges, and theoretical gaps remain. Future work should explore whether functional reserve from different cognitive domains similarly mitigates DTCs. Additionally, the low prevalence of SWWT (2.8%) limits generalizability. SWWT was recorded as ecological observations during conversation, which may have underestimated its occurrence or introduced observer bias. Standardizing SWWT assessment and longitudinal validation would help to clarify its role as a compensatory strategy or a marker of decline.

Sample characteristics further constrain generalizability. Our cohort was relatively healthy and more educated than the general older adult population, which may have introduced low SWWT prevalence and restricted range of functional reserve. Specifically, high-performing individuals may have been limited by the operational definition of ‘walking’ rather than their true physiological capacity. Moreover, SWWT may be more common in patients with stroke or dementia [25, 26], where dual-task capacity is more limited. While MoCa and MMSE scores were used to exclude cognitive impairment, future studies should include broader cognitive assessments. It would also be interesting to access potential cognitive decline across a border age range, such as middle age population [62].

Additionally, the counter intuitive positive cohort effect (higher cognitive and motor DTCs in recent cohorts) contradicts assumptions of generational improvements in mobility. While we residualized cohort on age to control for collinearity, unmeasured secular trends may confound this finding. Disentangle cohort and age effects using longitudinal designs would better clarify these generational differences.

Finally. task-specific limitations highlight opportunities for refinement. Our semantic (animal naming) and working memory (counting backwards) tasks may not fully capture cognitive load. Semantic network analyses or executive function tests could better quantify cognitive capacity. Similarly, while gait speed is a robust marker of mobility, spatiotemporal parameters (e.g., stride variability, step length) may reveal subtler dual-task effects. We are currently analysing these data for future work.

## Supplementary Information

Supplementary Material 1.

## Data Availability

The datasets used and/or analysed during the current study are available from the corresponding author on reasonable request.

## Abbreviations

DTC: Dual Task Cost
SWWT: Stops Walking when Talking
(G)LMM: Generalized Linear Mixed Model
BMC: Basel Mobility Center
MMSE: Mini-Mental State Examination
MoCA: Montreal Cognitive Assessment
M: Mean
SD: Standard Deviation
SEM: Standard Error of Mean
CI: Confidence Interval
AIC: Akaike Information Criterion
BIC: Bayesian Information Criterion
VIF: Variance Inflation Factor
cDTE: Combined dual-task effect
mAAI: Modified attention allocation index
