# Supplementary material for: Functional reserve mitigates cognitive-motor dual-task costs in older adults: insights from age, cohort, and behavioural strategies
Source: Eur Rev Aging Phys Act. 2026 Jun 25;23:24. doi: 10.1186/s11556-026-00423-z (PMC13326005; doi:10.1186/s11556-026-00423-z)
Supplement: Supplementary file 1 — Supplementary Material 1. [file 11556_2026_423_MOESM1_ESM.docx]

Supplemental Materials

# S1. Inclusion and exclusion criteria of the individual studies

All data were collected by the BMC members or the in-house Memory Clinic employees for routine clinical assessment or for different studies. Visitors who completed a gait analysis at the BMC were included. Visitors were excluded in case they did not consent with the scientific use of their data (i.e. rejected the “Generalkonsent Felix Platter”).

**Table S1.** Recruitment source of individual studies

| Study Name | Size of Sample Pooled to Current Study |
| --- | --- |
| BASEL | 889 |
| DO-HEALTH Cohort | 1 |
| Ginkgo | 10 |
| JDR Pilot | 94 |
| LYRA | 3 |
| MoCha Pilot | 4 |
| Studies before 2017 | 236 |
| NUDAL | 91 |
| SANDx | 23 |

**DO-HEALTH (Vitamin D3 - Omega3 - Home Exercise - Healthy Ageing and Longevity Trial) (2012–2018)**

**Inclusion Criteria:**

- Age ≥ 70 years.
- Mini Mental State Examination (MMSE) score ≥ 24.
- Community-dwelling.
- Sufficient mobility to attend study center visits.
- Ability to walk 10 meters (with/without walking aid) and transfer independently (e.g., in/out of a chair).
- Ability to swallow study capsules.
- Willingness to provide informed consent and comply with study procedures.

**Exclusion Criteria:**

- Vitamin D intake > 1000 IU/day in the past 36 months or a bolus of ≥ 300,000 IU in the past 12 months (unless willing to limit intake to 800 IU/day during the trial).
  - *Provision 1:* If intake was 1000–2000 IU/day in the past 3 months, a 3-month washout (≤ 800 IU/day) is required.
  - *Provision 2:* If intake was > 2000 IU/day, a 6-month washout is required.
- Unwillingness to limit calcium supplements to ≤ 500 mg/day.
- Use of omega-3 supplements in the past 3 months (unless willing to discontinue).
- Current use of active vitamin D metabolites (e.g., Rocaltrol, alphacalcidiol), PTH therapy (e.g., Teriparatide), or Calcitonin (unless willing to discontinue).
- Participation in another clinical trial in the past 4 months or planned participation in the next 3 years.
- Diagnosed in the past 5 years with:
  - Cancer (except non-melanoma skin cancer).
  - Myocardial infarction, stroke, transient ischemic attack, angina pectoris, or coronary artery intervention.
- Severe renal impairment (creatinine clearance ≤ 15 mL/min) or dialysis.
- Hypercalcemia (> 2.6 mmol/L).
- Hemiplegia or severe gait impairment.
- History of hypo-/hyperparathyroidism.
- Severe liver disease.
- Granulomatous diseases (e.g., tuberculosis, sarcoidosis).
- Major visual/hearing impairment or serious illness precluding participation.
- Household member already enrolled in DO-HEALTH.
- Residence in assisted living or nursing home.
- Acute fracture in the past 6 weeks (temporary exclusion).
- Epilepsy or use of anti-epileptic drugs.
- 3 falls in the past month.
- Paget’s disease (osteodystrophia deformans).
- *Germany-specific:* Institutionalization/imprisonment by court order (§40, Abs. 1, Art. 4, AMG).

**DO-HEALTH-C (Vitamin D3 - Omega3 - Home Exercise - Healthy Aging and Longevity Cohort) (2019–ongoing)**

**Inclusion Criteria:**

- Former participant in the DO-HEALTH clinical trial.

**Exclusion Criteria:**

- Inability/unwillingness to provide written informed consent.
- Medical conditions rendering assessments unreliable, burdensome, or unsafe.

**Study Population:**

- Former DO-HEALTH trial participants (NCT01745263).

**MOBITEC-STROKE (Recovery of Mobility Function and Life-Space Mobility After Ischemic Stroke) (2019–2022)**

**Inclusion Criteria:**

- Age ≥ 18 years.
- First ischemic stroke within the past 3 months.
- Ability to communicate verbally and provide written informed consent.
- Presence of ≥ 1 stroke-related symptom:
  - Lower limb paresis/ataxia.
  - Stance/gait ataxia (cerebellar or sensory).
  - Visual disturbance/field defect.
  - Central vestibular deficit.
  - Attentional deficit/neglect.
- Ability to rise from a chair and sit down without assistance.
- Ability to walk ≥ 20 meters (with/without walking aid or pauses, but without physical assistance).

**Exclusion Criteria:**

- Inability to walk without assistance.
- Wheelchair-bound or permanently bedridden (modified Rankin Scale > 3).
- Severe cognitive impairment (MoCA < 21 or < 20 for ≤ 12 years of education).
- Acute psychiatric disorder (e.g., severe depression).
- Advanced terminal illness.
- Orthopedic surgery of the lower extremities in the past year.
- Ongoing rehabilitation post-surgical procedure at stroke onset.
- Major pre-stroke mobility limitations (self-reported).
- *Note:* Use of centrally acting drugs is documented but not exclusionary.

**GINKGO-Study (Effect of Ginkgo Biloba Special Extract LI 1370 on Dual-Tasking in Patients with MCI) (2010–2015)**

**Inclusion Criteria:**

- Age 50–85 years.
- Swiss German or German speaker.
- Completed elementary school.
- Impaired executive function (gait speed reduction ≥ 10% under dual-task vs. normal walking).
- No dementia (per ICD-10/DSM-IV criteria).
- Cognitive decline (self/informant report or objective task).
- Preserved basic activities of daily living; minimal impairment in complex instrumental functions.
- Written informed consent.

**Exclusion Criteria:**

- Current treatment with warfarin-like drugs (e.g., Coumarins, Clopidogrel; acetylsalicylic acid ≤ 300 mg permitted).
- Ginkgo biloba extract (GBE) intake in the past 6 months.
- Hypersensitivity to GBE or its constituents.
- Regular use of antipsychotics, anxiolytics, or sedatives (unless clinically stable for ≥ 3 months).
- Gait-relevant disorders:
  - Severe cardio/pulmonary/cerebrovascular disorders.
  - Bleeding diathesis.
  - Polyneuropathy.
  - Severe orthopedic disorders.
  - Organic cerebral disease.
  - Epilepsy.
  - Low vision.
- Severe medical conditions (e.g., chronic renal insufficiency, severe hepatic disorders, uncontrolled hypertension, peptic ulcer, malignancy).
- Participation in another clinical intervention study in the past 2 months.
- Use of walking aids.
- Normal walking speed < 100 cm/s.

**fMRI Study (Imaging Gait Analysis: An fMRI Dual-Task Study) (2016–2017)**

**Inclusion Criteria:**

- Younger volunteers (mean age 27.9 ± 4.44 years; 9 females) or older volunteers ≥ 70 years (mean age 75.83 ± 4.27 years; 14 females).
- Self-reported as healthy.
- Right-handed (per Annett’s handedness test).
- Written informed consent.

**Exclusion Criteria:**

- History of neurological or psychiatric disorders.
- Lack of written informed consent.

**SANDx (Effects of the SGLT2-Inhibitor Empagliflozin on Patients with Chronic SIADH) (2017–2021)**

**Inclusion Criteria:**

- Age ≥ 18 years.
- Hyponatremia (< 133 mmol/L) due to chronic (> 72 hours) SIADH, defined as:
  - Serum osmolality < 275 mOsm/kg.
  - Urine osmolality > 100 mOsm/kg.
  - Urine sodium > 30 mmol/L.

**Exclusion Criteria:**

- Acute (< 72 hours) or transient hyponatremia.
- Severe symptomatic hyponatremia requiring hospitalization.
- Type 1 diabetes mellitus.
- Uncontrolled hypothyroidism or adrenal insufficiency.
- Renal impairment (GFR < 45 mL/min).
- Cardiac failure.
- Symptomatic liver disease/severe hepatic impairment (ALT/AST > 3× upper limit).
- Treatment with SGLT2 inhibitors, lithium chloride, urea, or glitazones.
- Severe immunosuppression.
- Pregnancy or breastfeeding.
- Palliative/end-of-life care.

**PCS (Power Centering for Seniors: A Multimodal Intervention to Improve Mobility and Quality of Life in Older Adults) (2021–2022)**

**Inclusion Criteria:**

- Age ≥ 70 years.
- Community-dwelling.
- Ability to walk ≥ 5 meters (with/without walking aid, but without assistance).
- Normal walking speed: 80–100 cm/s.
- MoCA score ≥ 18.
- Written informed consent.

**Exclusion Criteria:**

- Clinically significant neurologic/musculoskeletal diseases severely affecting walking (e.g., advanced Parkinson’s disease, hemiplegia).
- Unstable medical/psychiatric conditions (e.g., renal failure, hepatic dysfunction, cardiovascular disease, advanced COPD, psychosis) that may endanger the participant or affect adherence.
- Terminal illness.
- Fracture (except dental) in the past 3 months.
- Blindness.
- Inability to follow study procedures (e.g., language barriers, dementia).
- Prior participation in the Power Centering for Seniors program.
- Current participation in another non-observational clinical study.

**NUDAL (Effects of Daily Nutritional Supplementation in Combination with Eurhythmics Training) (2012–2014)**

**Inclusion Criteria:**

- Community-dwelling males/females ≥ 65 years.
- Ability to walk 15 meters unassisted.
- MMSE ≥ 24.
- Written informed consent.

**Exclusion Criteria:**

- Serum 25(OH) vitamin D > 100 nmol/L at screening.
- Regular (> 3×/week) use of high-protein oral nutritional supplements.
- Milk protein allergy (lactose intolerance).
- Severe visual impairment (corrected near vision < 0.2 in both eyes).
- Severe neurologic/orthopedic/rheumatologic/psychiatric illness precluding task compliance or unassisted walking.
- Terminal illness with life expectancy < 12 months.

**LYRA-Study (Effect of Lyra Gait Training on the Mobility of Geriatric Rehabilitation Inpatients) (2017–2018)**

**Inclusion Criteria:**

- Age ≥ 65 years.
- Rehabilitation inpatients at Felix Platter Hospital with primary muscle weakness and walking difficulty.
- Community-dwelling pre-admission; planned discharge home.
- Body weight ≤ 150 kg; height 100–195 cm.
- MMSE ≥ 21.
- Written informed consent.

**Exclusion Criteria:**

- Clinically significant neurologic/musculoskeletal diseases severely affecting walking (e.g., advanced Parkinson’s disease, hemiplegia).
- Severe cardiac insufficiency.
- Advanced COPD (Gold IV).
- Amputations (except fingers).
- Blindness.
- Inability to understand/speak German sufficiently.

**Pilot Study of Virtual Gait Training in Older Adults (2011)**

**Inclusion Criteria:**

1. Age > 65 years.
2. Fall within the past 6 months.

**Exclusion Criteria:**

- Cognitive deficits.
- Physical disability.

**Basel Study on the Elderly (BASEL) (1997–2013)**

**Inclusion Criteria:**

- Surviving participants of the preceding *Basel Longitudinal Study on Aging (1955–1978)*.
- Original sample (1955–1978): 123 males (Ciba-Geigy employees: apprentices, technicians, academicians, management; and children from the Basel area).

# S2. Standard Operating Procedure (SOP)


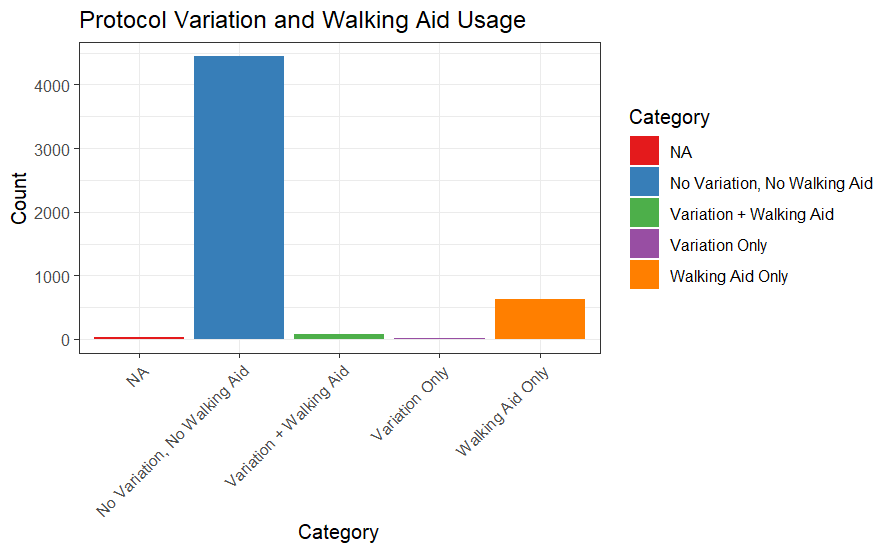


**Figure S1.** Frequency of Protocol Variation and Walking Aids Usage. Note that only when nether variation in protocol nor walking aids used, then the data is included in the current study.


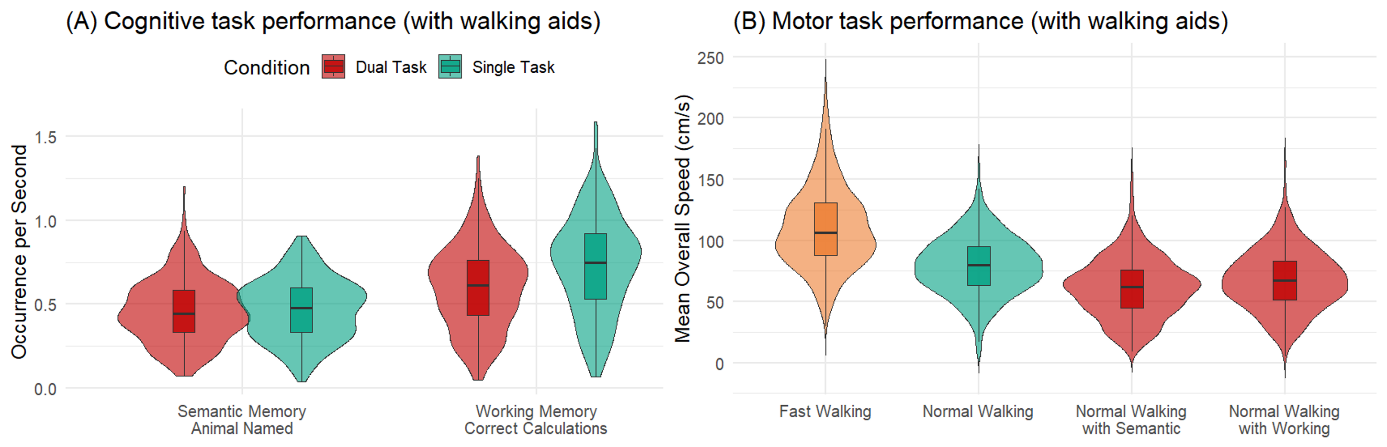


**Figure S2.** Cognitive and motor task performance of older adults with walking aids. They show similar patterns in cognitive domain and dual-task interference, whereas their speed was generally slower than participants without walking aids in all conditions.

# SOP§2.5 Procedure of the Examination with the Patient

The sequence of walks (synonym: tests or trials) is to be conducted in accordance with the examination form. Each task is explained to the patient beforehand. Once the patient has no further questions, the test is initiated in the GAITRite software. The test administrator positions themselves slightly behind the chair and asks the patient to stand up, remaining ready to assist if necessary.

After receiving the respective walking instructions from the test administrator, the patient performs the task.

The patient should either sit down or remain standing in front of the chair between tasks. After each task, the test administrator returns to the workstation, saves the current trial in GAITRite, and prepares the next test. If the task is performed correctly, it is checked off on the examination form.

**Note:** If the patient can only walk 12 meters with difficulty or tires quickly, the gait analysis is conducted using a second chair placed at the end of the walkway (at the end of the extension). The instruction is then not *“beyond the walkway”* (“bis über den Teppich hinaus”), but *“to the chair”* (“bis zum Stuhl”). This is subsequently recorded in Filemaker under *“Variation of Standard Protocol”* (“Variation of Standard protocol”).

Continue with the instructions for the next task.

Instructions before the gait analysis

“You will walk across this carpet four to five times (pointing to the walkway), each time with a different task. I will tell you the task each time. I will walk with you every time.”

Instruction: Normal Walking

“When I say ‘Go’, walk normally, beyond the carpet. Ready, set, go.”
(„Wenn ich ‚Los’ sage, gehen Sie ganz normal, bis über den Teppich hinaus. Achtung, fertig, los.“)

Instruction: Slow Walking (discontinued since 01/2023, not included in current studies)

“This time, when I say ‘Go’, walk slowly, beyond the carpet. Slower than normal. Ready, set, go.”
(„Dieses Mal, wenn ich ‚Los’ sage, gehen Sie langsam, bis über den Teppich hinaus. Langsamer als normal. Achtung, fertig, los.“)

Instruction: Fast Walking

“This time, when I say ‘Go’, walk as fast as possible, beyond the carpet. Do not run or jump—just walk as fast as possible. Ready, set, go.”
(*„Dieses Mal, wenn ich ‚Los’ sage, gehen Sie so schnell wie möglich, bis über den Teppich hinaus. Nicht rennen oder springen, so schnell wie möglich gehen. Achtung, fertig, los.“*)

Instruction: Working Memory Task (while walking)

“This time, walk normally and simultaneously count backward aloud. Start at 50 and subtract 2 each time. Ready, set, go.”
(„Dieses Mal gehen Sie ganz normal und gleichzeitig laut Rückwärtsrechnen. Mit 50 beginnen und immer 2 abziehen. Achtung, fertig, los.“)

Instruction: Semantic Memory Task (while walking)

“This time, walk normally and simultaneously name animals aloud. Any kind of animals—whatever comes to mind. Ready, set, go.”
(„Dieses Mal gehen Sie ganz normal und gleichzeitig laut Tiere aufzählen. Egal was für Tiere, was Ihnen in den Sinn kommt. Achtung, fertig, los.“)

Instruction: Normal Walking with Walking Aid (if applicable; not included in current studies)

“This time, walk normally with your walking aid, beyond the carpet. Ready, set, go.”
(„Dieses Mal gehen Sie mit Ihrer Gehhilfe ganz normal, bis über den Teppich hinaus. Achtung, fertig, los.“)

Notes

**Note 1:**
Instructions vary in emphasis depending on the task. *“Ready, set”* (“Achtung, fertig”) is always spoken in the same manner. *“Go”* (“Los”) is spoken with higher frequency and faster for fast walking, and lower frequency and slower for slow walking. Adjust speech tempo and insert pauses according to the patient’s comprehension.

**Note 2:**
General observations and relevant patient information should be documented on the examination form next to each test or under *“Notes.”* Comments made by the patient should be transcribed verbatim in round brackets, e.g., (esch das langsam gnue), while comments by the test administrator are recorded in square brackets, e.g., [jo]. Observations should be noted without brackets, e.g., *last 7 footfalls deleted, too close to edge of walkway, 9 remaining.*

Post-01/2023 Protocol Update

Two additional tasks are conducted while seated. These are independent of GAITRite. The test administrator stands in front of the patient on the walkway extension.

**Pre-information:**
“The final two tasks will now be repeated while sitting.”

Instruction: Working Memory Task (while sitting)

“While sitting, count backward aloud. Start at 50 and subtract 2 each time. Ready, set, go.”
(*„Im Sitzen, laut Rückwärtsrechnen. Mit 50 beginnen und immer 2 abziehen. Achtung, fertig, los.“*)

Instruction: Semantic Memory Task (while sitting)

“While sitting, name animals. They can be the same as before, but do not have to be. Whatever comes to mind. Ready, set, go.”
(„Im Sitzen Tiere aufzählen. Es können die gleichen Tiere sein wie vorhin, muss aber nicht sein. Was Ihnen in den Sinn kommt. Achtung, fertig, los.“)

Conclusion

This concludes the gait analysis for the patient.

**Example phrasing:**
“Now we are finished. You may remain seated for a moment, and then I will accompany you to the medical examination.”

The test administrator transfers the task responses to the examination form and notes whether dynamic editing is required and whether any deviations from the standard protocol occurred.

# S3. Descriptive Statistics on cognitive and motor task performance

**Table S2.** Descriptive Statistics of Single- and Dual-Task Performance across Cognitive and Motor Domains.

| **Condition** | **n** | **mean** | **sd** | **median** | **trimmed** | **mad** | **min** | **max** | **range** | **skew** | **kurtosis** | **se** |
| --- | --- | --- | --- | --- | --- | --- | --- | --- | --- | --- | --- | --- |
| Single Semantic Memory | 1639 | 0.599 | 0.214 | 0.583 | 0.592 | 0.213 | 0.065 | 1.500 | 1.435 | 0.417 | 0.317 | 0.005 |
| Single Working Memory | 1761 | 0.584 | 0.201 | 0.583 | 0.580 | 0.206 | 0.028 | 1.556 | 1.528 | 0.344 | 0.611 | 0.005 |
| Dual Semantic Memory | 1629 | 0.908 | 0.276 | 0.917 | 0.910 | 0.258 | 0.012 | 2.125 | 2.113 | 0.010 | 0.511 | 0.007 |
| Dual Working Memory | 1716 | 0.747 | 0.239 | 0.750 | 0.747 | 0.222 | 0.010 | 1.600 | 1.590 | 0.089 | 0.352 | 0.006 |
| Normal Walking | 4438 | 115.938 | 21.863 | 116.300 | 116.172 | 21.201 | 28.600 | 204.200 | 175.600 | -0.092 | 0.239 | 0.328 |
| Fast Walking | 4315 | 164.078 | 33.678 | 163.900 | 163.893 | 32.765 | 42.700 | 333.000 | 290.300 | 0.164 | 0.653 | 0.513 |
| Dual Walking&Semantic Memory | 4271 | 87.691 | 28.464 | 88.700 | 88.154 | 27.725 | 5.700 | 179.600 | 173.900 | -0.118 | -0.044 | 0.436 |
| Dual Walking&Working Memory | 4274 | 96.950 | 27.760 | 97.500 | 97.099 | 26.835 | 5.800 | 190.100 | 184.300 | -0.040 | 0.082 | 0.425 |

**Table S3.** Descriptive Statistics of Single- and Dual-Task Performance across Cognitive and Motor Domains (exclude data with missing values).

| **Condition** | **n** | **mean** | **sd** | **median** | **trimmed** | **mad** | **min** | **max** | **range** | **skew** | **kurtosis** | **se** |
| --- | --- | --- | --- | --- | --- | --- | --- | --- | --- | --- | --- | --- |
| Single Semantic Memory | 1561 | 0.604 | 0.210 | 0.583 | 0.598 | 0.213 | 0.065 | 1.500 | 1.435 | 0.376 | 0.220 | 0.005 |
| Single Working Memory | 1561 | 0.587 | 0.196 | 0.583 | 0.582 | 0.194 | 0.028 | 1.556 | 1.528 | 0.312 | 0.561 | 0.005 |
| Dual Semantic Memory | 1561 | 0.914 | 0.267 | 0.917 | 0.915 | 0.258 | 0.012 | 2.125 | 2.113 | 0.067 | 0.507 | 0.007 |
| Dual Working Memory | 1561 | 0.750 | 0.238 | 0.750 | 0.749 | 0.222 | 0.010 | 1.600 | 1.590 | 0.087 | 0.343 | 0.006 |
| Normal Walking | 1561 | 116.610 | 21.086 | 117.200 | 116.747 | 20.905 | 36.700 | 194.200 | 157.500 | -0.071 | 0.069 | 0.534 |
| Fast Walking | 1561 | 166.736 | 34.335 | 166.700 | 166.656 | 33.507 | 42.700 | 333.000 | 290.300 | 0.180 | 0.721 | 0.869 |
| Dual Walking&Semantic Memory | 1561 | 87.639 | 27.407 | 88.600 | 88.094 | 27.132 | 7.600 | 179.600 | 172.000 | -0.119 | -0.032 | 0.694 |
| Dual Walking&Working Memory | 1561 | 97.085 | 27.135 | 98.500 | 97.286 | 25.501 | 7.900 | 186.900 | 179.000 | -0.049 | 0.254 | 0.687 |


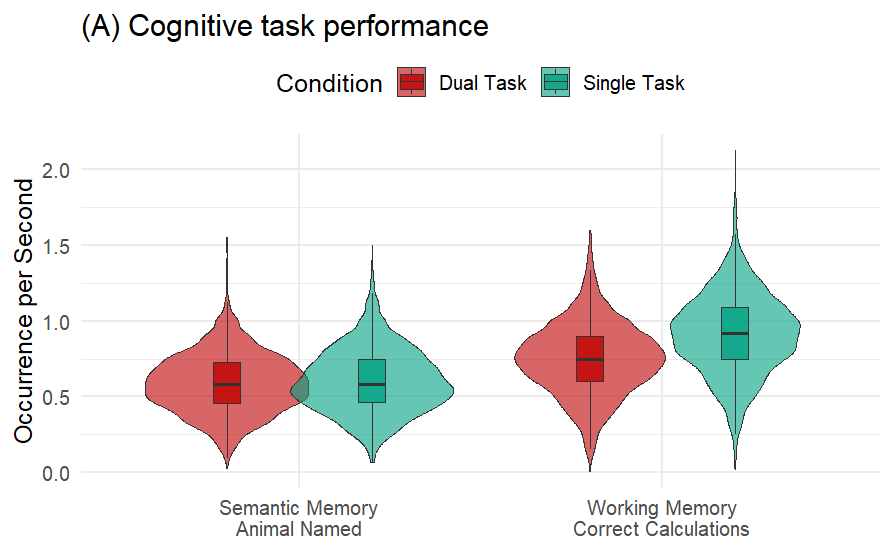

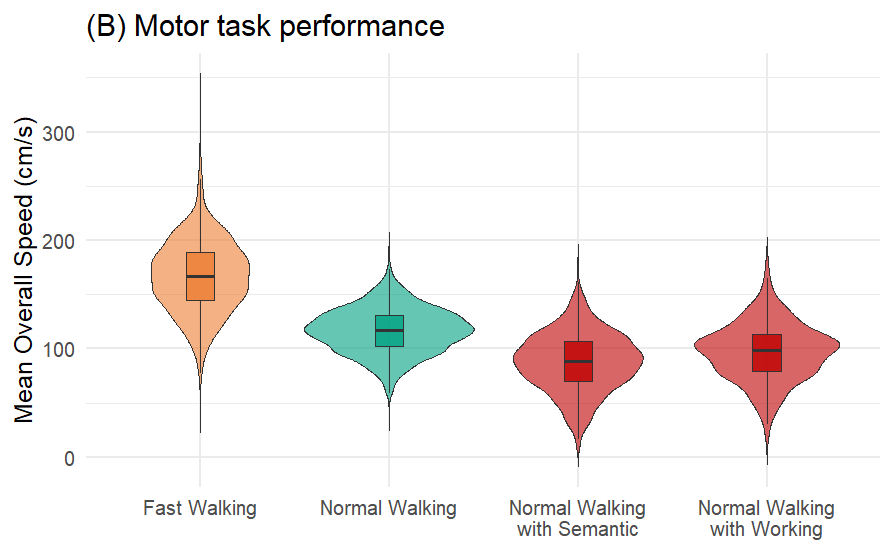


**Figure S3.** Cognitive and motor task performance with only complete data. They show similar patterns in cognitive domain and dual-task interference.

# S4. Linear Mixed Modelling Protocol

- 1. Dependent variables: Dual-task costs. motor cost - drop of speed when dual-tasking, cognitive cost - drop of count per second when dual-tasking. Fall Risk - probability of having fall history
  2. Independent variables:

For cognitive and motor costs - LMM

Age (z score)

Cohort (z score) - birth cohort as test date minus age

Cognitive Task Type (semantic vs working memory)

Functional Reserve (z score)

Stop walking when talking (yes, no)

Fall History(yes, no)

Fall Fear (yes, no)

For fall risk - GLMM

Fall Fear (yes, no)

Age (z score)

Cohort (z score)

Dual task costs (z score)

Single task costs (z score)

Functional Reserve (z score)

Stop walking when talking(yes, no)

- 1. Work Flow:
     - Full model with random intercepts - variances
     - Full model with random intercepts and random slopes
     - [if residual plot shows cluster, then split by, for example, cognitive task type]
     - if not converge:
     - drop random effect correlations
     - drop unimportant fix effects
     - drop one by one random slopes (only kept ID as random effect for motor. Details below, we need to be careful with interpretation.)
     - Check overfitting and robustness
     - If a random effect variance is near zero, remove it.
     - If fixed effect p-values are very high, remove them.
     - If random intercept and slope correlation is >|0.8|, remove correlation.

# S5. Full Multilevel Model Function

## DTC models:

- $\boldsymbol{\delta}_{\boldsymbol{ij}}\boldsymbol{=}\boldsymbol{\beta}_{\boldsymbol{0}}\boldsymbol{+}\boldsymbol{\beta}_{\boldsymbol{k}}\boldsymbol{X}_{\boldsymbol{k}}\boldsymbol{+}\boldsymbol{u}_{\boldsymbol{0}\boldsymbol{j}}\boldsymbol{+}\boldsymbol{u}_{\boldsymbol{kj}}\boldsymbol{X}_{\boldsymbol{k}}\boldsymbol{+}\boldsymbol{\epsilon}_{\boldsymbol{ij}}$
- $\boldsymbol{X}_{\boldsymbol{k}}\boldsymbol{=}\boldsymbol{\{}\boldsymbol{Z}_{\boldsymbol{Cohor}\boldsymbol{t}_{\boldsymbol{ij}}}\boldsymbol{,SWW}\boldsymbol{T}_{\boldsymbol{ij}}\boldsymbol{,}\boldsymbol{Z}_{\boldsymbol{F}\boldsymbol{R}_{\boldsymbol{ij}}}\boldsymbol{,Fal}\boldsymbol{l}_{\boldsymbol{ij}}\boldsymbol{,Fea}\boldsymbol{r}_{\boldsymbol{ij}}\boldsymbol{, Tas}\boldsymbol{k}_{\boldsymbol{ij}}\boldsymbol{\}}$
- Dependent Variable
  - $\delta_{ij}\text{: Dual-task cost for subject }j\text{ at observation }i.$
    - $\delta_{motor_{ij}}\text{: Motor dual-task cost}\text{.}$
    - $\delta_{cognitive_{ij}}\text{: Cognitive dual-task cost}\text{.}$
- Fixed Effects
  - $\beta_{0}\text{: Fixed intercept (population-average baseline dual-task cost).}$
  - $\beta_{k}X_{k}\text{: Fixed effect of }k^{th}\text{predictor }\text{ (consistent across all subjects).}$
- Random Effects
  - $u_{0j}\sim N\left( 0,\sigma_{u0}^{2} \right)\text{: Subject-specific deviation from }\beta_{0}.$
  - $u_{kj}\sim N\left( 0,\sigma_{uk}^{2} \right)\text{: Subject-specific deviation in the effect of predictor }X_{k}$.
- Residual Error
  - $\epsilon_{ij}\sim N\left( 0,\sigma^{2} \right)\text{: Unexplained within-subject variance.}$

## Fall Models

$$\log\left( \frac{\boldsymbol{\pi}_{\text{ij}}}{\boldsymbol{1-}\boldsymbol{\pi}_{\text{ij}}} \right)\boldsymbol{=}\boldsymbol{\beta}_{\boldsymbol{0}}\boldsymbol{+}\boldsymbol{\beta}_{\boldsymbol{k}}\boldsymbol{X}_{\boldsymbol{k}}\boldsymbol{+}\boldsymbol{u}_{\text{0j}}\boldsymbol{+}\boldsymbol{u}_{\boldsymbol{1}\boldsymbol{j}}\boldsymbol{Z}_{\boldsymbol{\delta}_{\boldsymbol{ij}}}\boldsymbol{+}\boldsymbol{\epsilon}_{\text{ij}}$$

$$\boldsymbol{X}_{\boldsymbol{k}}\boldsymbol{=}\boldsymbol{\{}\boldsymbol{Z}_{\boldsymbol{Cohor}\boldsymbol{t}_{\text{ij}}}\boldsymbol{,SWW}\boldsymbol{T}_{\text{ij}}\boldsymbol{,}\boldsymbol{Z}_{\boldsymbol{F}\boldsymbol{R}_{\text{ij}}}\boldsymbol{,Fea}\boldsymbol{r}_{\text{ij}}\boldsymbol{,}\boldsymbol{Z}_{\boldsymbol{\delta}_{\boldsymbol{ij}}}\boldsymbol{,Domai}\boldsymbol{n}_{\boldsymbol{ij}}\boldsymbol{,Tas}\boldsymbol{k}_{\boldsymbol{ij}}\boldsymbol{\}}$$

$$\boldsymbol{\pi}_{\boldsymbol{ij}}\boldsymbol{\to probability of fall of individual i within group j}$$

# S6. Linear Mixed Modelling Prerequisites


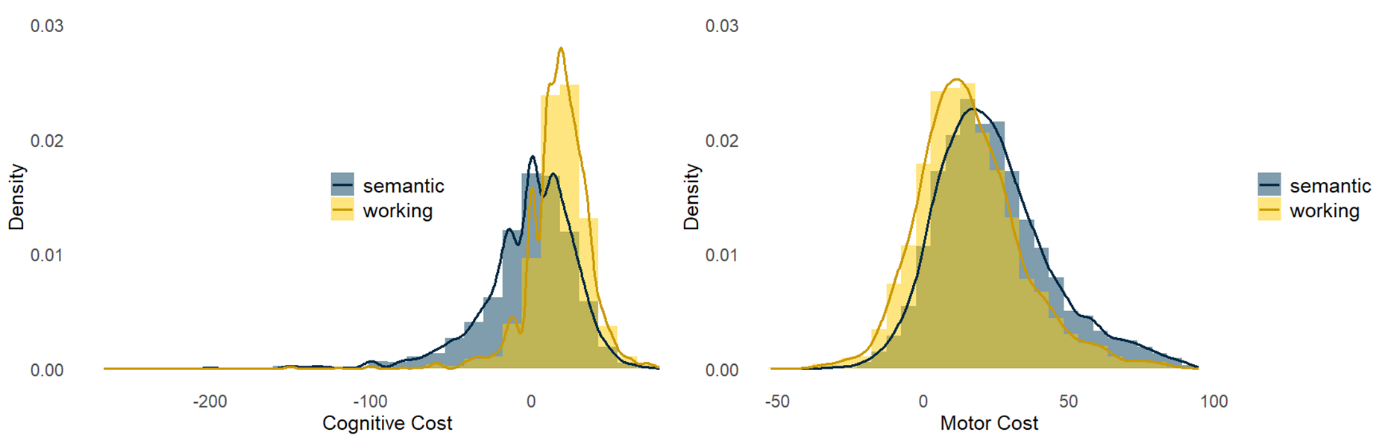


**Figure S4.** Distribution of Cognitive and Motor Dual-Task Costs.


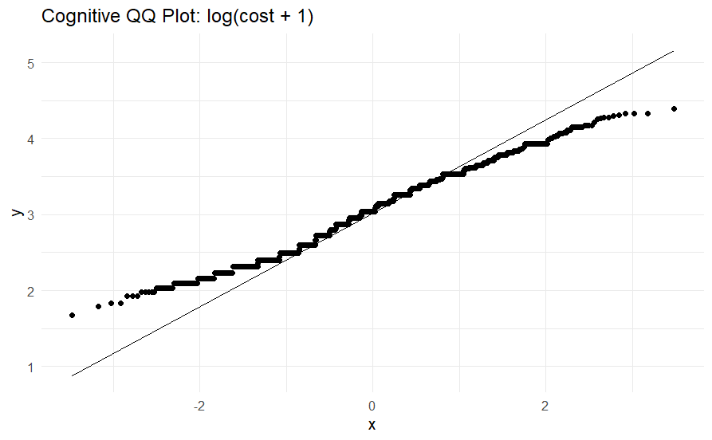

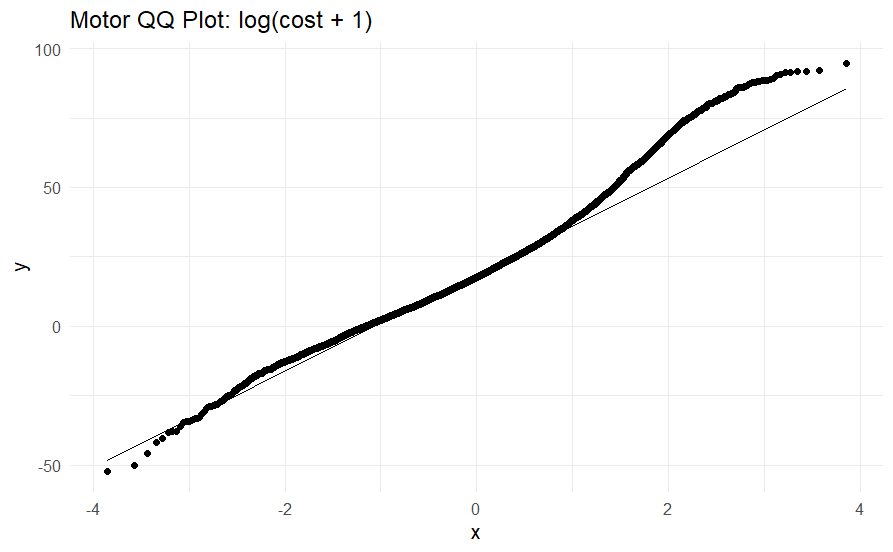


**Figure S5.** Q-Q Plots of Cognitive and Motor Dual-Task Costs after Log Transformed.


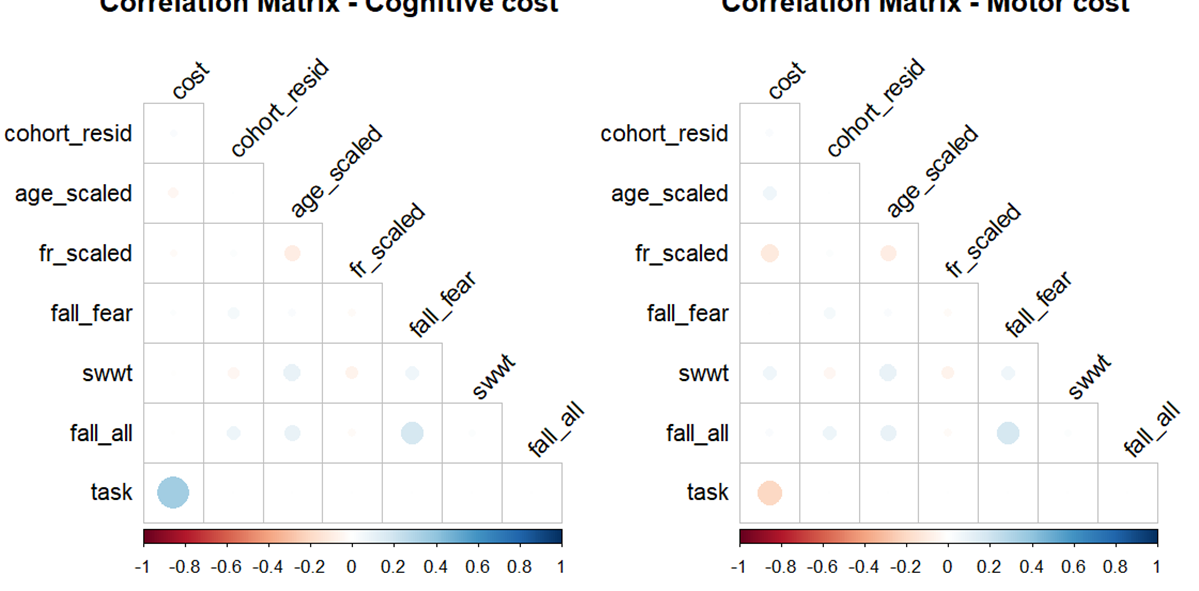


**Figure S6.** Correlation Plots of Cognitive and Motor Dual-Task Costs with Full Predictors.

**Table S4: Variance Inflation Factor (VIF) Values for Dual-Task Costs**

| Predictor | Motor Cost VIF | Cognitive Cost VIF |
| --- | --- | --- |
| cohort_resid | 1.014427 | 1.012798 |
| age_scaled | 1.028081 | 1.026994 |
| fr_scaled | 1.014393 | 1.026791 |
| fall_fear | 1.032984 | 1.025611 |
| swwt | 1.019768 | 1.015549 |
| fall_all | 1.043019 | 1.033011 |
| task | 1.000000 | 1.000299 |

# S7. Multilevel Model Complete Diagnosis

## Motor DTC

Linear mixed model fit by REML. t-tests use Satterthwaite's method ['lmerModLmerTest']

Formula: cost_log ~ age_scaled + cohort_resid + fr_scaled + task + fall_fear + swwt + fall_all + (1 | id)

Data: motor_data

REML criterion at convergence: 15948.2

Scaled residuals:

Min 1Q Median 3Q Max

-7.3953 -0.3302 0.1072 0.4831 3.2190

Random effects:

Groups Name Variance Std.Dev.

id (Intercept) 0.5483 0.7404

Residual 0.3898 0.6243

Number of obs: 6311, groups: id, 2888

Fixed effects:

Estimate Std. Error df t value Pr(>|t|)

(Intercept) 2.884e+00 4.103e-02 4.225e+03 70.289 < 2e-16 ***

age_scaled 3.687e-02 1.563e-02 2.936e+03 2.359 0.01838 *

cohort_resid 8.551e-02 2.877e-02 3.388e+03 2.972 0.00298 **

fr_scaled -1.024e-01 1.479e-02 4.136e+03 -6.925 5.05e-12 ***

task -1.849e-01 8.001e-03 3.309e+03 -23.112 < 2e-16 ***

fall_fear -1.093e-02 2.202e-02 4.890e+03 -0.496 0.61958

swwt 8.680e-02 3.726e-02 4.775e+03 2.330 0.01985 *

fall_all -1.750e-02 1.442e-02 4.777e+03 -1.214 0.22466

---

Signif. codes: 0 ‘***’ 0.001 ‘**’ 0.01 ‘*’ 0.05 ‘.’ 0.1 ‘ ’ 1

Correlation of Fixed Effects:

(Intr) ag_scl chrt_r fr_scl task fll_fr swwt

age_scaled -0.064

cohort_resd -0.078 -0.053

fr_scaled 0.073 0.085 -0.024

task 0.009 0.003 0.001 0.017

fall_fear 0.370 0.002 -0.029 0.007 0.000

swwt 0.815 -0.076 0.060 0.063 -0.006 -0.046

fall_all -0.014 -0.077 -0.109 0.006 0.003 -0.134 -0.014

age_scaled cohort_resid fr_scaled task fall_fear fall_all swwt

1.023818 1.021166 1.013331 1.000351 1.022836 1.039609 1.016738

Computing bootstrap confidence intervals ... 2.5 % 97.5 %

.sig01 0.710352227 0.76628024

.sigma 0.609365935 0.63985861

(Intercept) 2.796343578 2.96417170

age_scaled 0.004992552 0.06648370

cohort_resid 0.022375607 0.14238842

fr_scaled -0.130544144 -0.07201988

task -0.201401153 -0.16853503

fall_fear -0.055778233 0.03014948

fall_all -0.049796830 0.01187286

swwt 0.011108132 0.15942063

# Fixed Effects

Parameter | Coefficient | SE | 95% CI | t(6301) | p

-------------------------------------------------------------------------

(Intercept) | -0.03 | 0.02 | [-0.06, 0.01] | -1.57 | 0.117

age scaled | 0.04 | 0.02 | [ 0.01, 0.07] | 2.36 | 0.018

cohort resid | 0.05 | 0.02 | [ 0.02, 0.08] | 2.97 | 0.003

fr scaled | -0.11 | 0.02 | [-0.14, -0.08] | -6.92 | < .001

task | -0.19 | 8.33e-03 | [-0.21, -0.18] | -23.11 | < .001

fall fear | -7.28e-03 | 0.01 | [-0.04, 0.02] | -0.50 | 0.620

fall all | -0.02 | 0.01 | [-0.05, 0.01] | -1.21 | 0.225

swwt | 0.03 | 0.01 | [ 0.01, 0.06] | 2.33 | 0.020

# Random Effects

Parameter | Coefficient

--------------------------------

SD (Intercept: id) | 0.77

SD (Residual) | 0.65

Uncertainty intervals (equal-tailed) and p-values (two-tailed) computed using a Wald t-distribution

approximation.Observations: 6311

Marginal R²: 0.05 Conditional R²: 0.605

Deviance: 15948.22


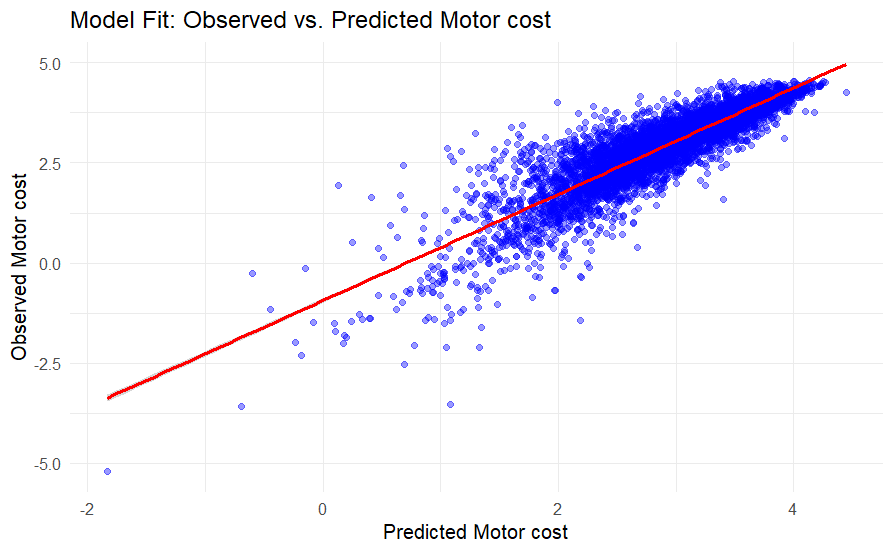


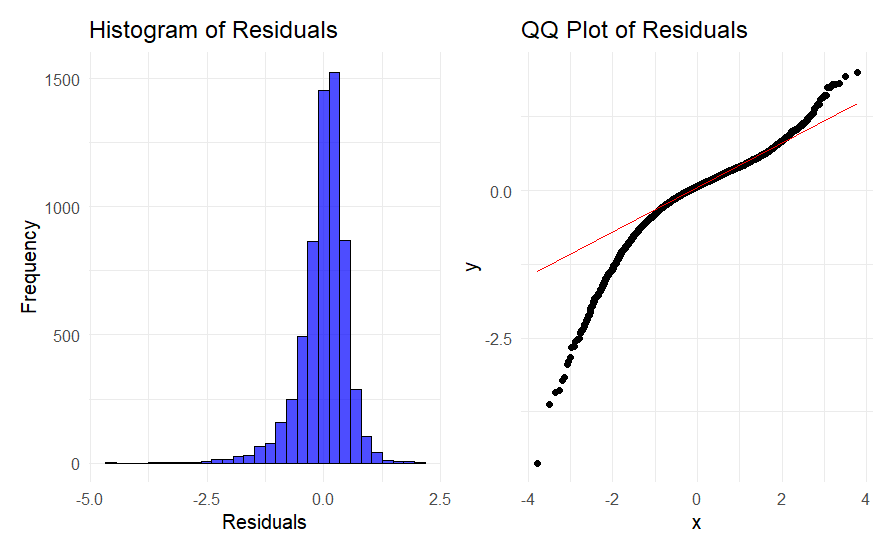


## Cognitive DTC

Linear mixed model fit by REML. t-tests use Satterthwaite's method ['lmerModLmerTest']

Formula: cost_log ~ age_scaled + cohort_resid + task + fr_scaled + fall_fear + swwt + fall_all + (1 | id)

Data: cog_data

REML criterion at convergence: 2452.2

Scaled residuals:

Min 1Q Median 3Q Max

-2.69374 -0.76560 0.02058 0.75180 2.62140

Random effects:

Groups Name Variance Std.Dev.

id (Intercept) 0.009399 0.09695

Residual 0.234483 0.48423

Number of obs: 1689, groups: id, 1155

Fixed effects:

Estimate Std. Error df t value Pr(>|t|)

(Intercept) 3.060e+00 4.235e-02 1.108e+03 72.270 < 2e-16 ***

age_scaled -8.019e-03 1.195e-02 1.036e+03 -0.671 0.50241

cohort_resid 6.867e-02 2.704e-02 1.162e+03 2.540 0.01122 *

task 2.195e-02 1.234e-02 1.090e+03 1.778 0.07566 .

fr_scaled -3.400e-02 1.231e-02 1.100e+03 -2.761 0.00585 **

fall_fear 1.757e-02 1.804e-02 1.109e+03 0.974 0.33046

swwt 3.949e-02 3.854e-02 1.056e+03 1.025 0.30572

fall_all 6.822e-03 1.235e-02 1.079e+03 0.552 0.58074

---

Signif. codes: 0 ‘***’ 0.001 ‘**’ 0.01 ‘*’ 0.05 ‘.’ 0.1 ‘ ’ 1

Correlation of Fixed Effects:

(Intr) ag_scl chrt_r task fr_scl fll_fr swwt

age_scaled -0.063

cohort_resd -0.315 0.042

task -0.078 -0.013 0.018

fr_scaled 0.069 0.128 -0.009 -0.020

fall_fear 0.286 0.040 -0.043 0.007 0.038

swwt 0.830 -0.080 0.048 0.007 0.076 -0.045

fall_all -0.013 -0.059 -0.091 -0.016 0.014 -0.132 -0.008

# Fixed Effects

Parameter | Coefficient | SE | 95% CI | t(1679) | p

----------------------------------------------------------------------

(Intercept) | -2.44e-04 | 0.02 | [-0.05, 0.05] | -9.95e-03 | 0.992

age scaled | -0.02 | 0.02 | [-0.07, 0.03] | -0.67 | 0.502

cohort resid | 0.06 | 0.02 | [ 0.01, 0.11] | 2.54 | 0.011

fr scaled | -0.07 | 0.02 | [-0.12, -0.02] | -2.76 | 0.006

task | 0.04 | 0.02 | [ 0.00, 0.09] | 1.78 | 0.076

fall fear | 0.02 | 0.02 | [-0.02, 0.07] | 0.97 | 0.330

fall all | 0.01 | 0.02 | [-0.04, 0.06] | 0.55 | 0.581

swwt | 0.03 | 0.02 | [-0.02, 0.07] | 1.02 | 0.306

# Random Effects

Parameter | Coefficient

--------------------------------

SD (Intercept: id) | 0.20

SD (Residual) | 0.98

Uncertainty intervals (equal-tailed) and p-values (two-tailed) computed using a Wald t-distribution

approximation.Observations: 1689

Marginal R²: 0.012 Conditional R²: 0.05

Deviance: 2452.221


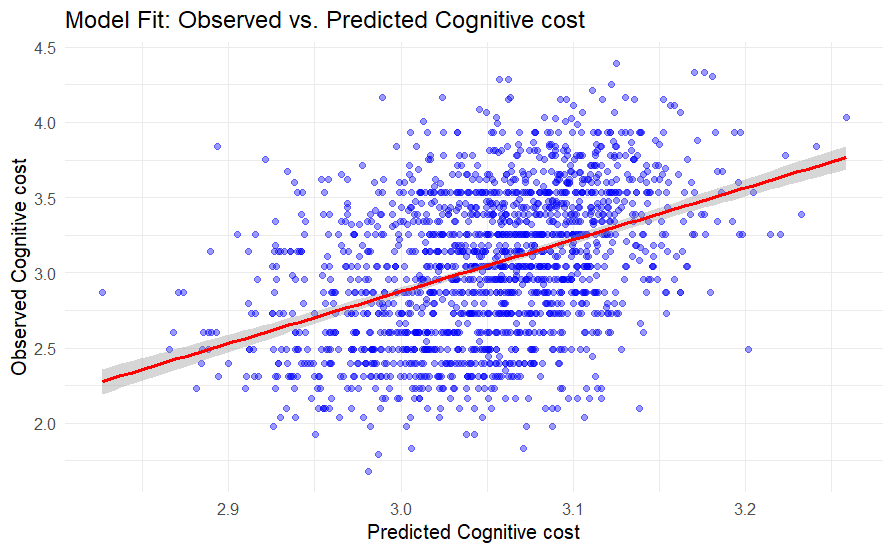


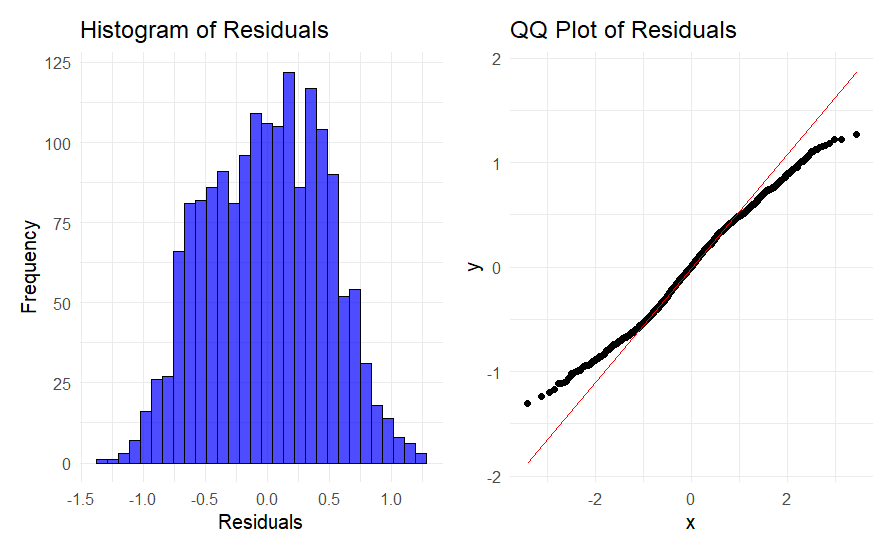


## Fall History

Model Summary

Generalized Linear Mixed Model (GLMM) Fit by Maximum Likelihood (Laplace Approximation)

Family: Binomial (logit link)

Formula:
fall_all ~ cohort_resid + age_scaled + fr_scaled + swwt + cost_scaled + fall_fear + (1 | id)

Data: fall_data_long

Optimizer: bobyqa

Model Fit Statistics:

| AIC | BIC | logLik | -2*log(L) | df.resid |
| --- | --- | --- | --- | --- |
| 6142.4 | 6199.9 | -3063.2 | 6126.4 | 9802 |

Residuals

Scaled Residuals:

| Min | 1Q | Median | 3Q | Max |
| --- | --- | --- | --- | --- |
| -3.264 | -0.006 | -0.003 | 0.066 | 3.152 |

Random Effects

| Groups | Name | Variance | Std.Dev. |
| --- | --- | --- | --- |
| id | (Intercept) | 922.3 | 30.37 |

Number of Observations: 9,810
Number of Groups (id): 3,025

Fixed Effects

| Parameter | Log-Odds | SE | 95% CI | z | p |
| --- | --- | --- | --- | --- | --- |
| (Intercept) | -10.26 | 0.24 | [-10.74, -9.79] | -42.36 | < .001 |
| cohort_resid | 0.47 | 0.13 | [0.21, 0.73] | 3.57 | < .001 |
| age_scaled | 0.51 | 0.15 | [0.21, 0.80] | 3.36 | < .001 |
| fr_scaled | 0.18 | 0.11 | [-0.04, 0.41] | 1.59 | 0.112 |
| swwt | 0.005 | 0.09 | [-0.17, 0.18] | 0.06 | 0.951 |
| cost_scaled | -0.11 | 0.08 | [-0.27, 0.05] | -1.37 | 0.170 |
| fall_fear | 0.39 | 0.11 | [0.18, 0.61] | 3.57 | < .001 |

Notes:

Uncertainty intervals (95% CI) and p-values computed using a Wald z-distribution approximation.

Random Effects

| Parameter | Coefficient |
| --- | --- |
| SD (Intercept: id) | 30.37 |

Model Performance

Observations: 9,810

Marginal R²: 0.001

Conditional R²: 0.996

Deviance: 1129.747

Correlation of Fixed Effects

|  | (Intr) | cohort_resid | age_scaled | fr_scaled | swwt | cost_scaled |
| --- | --- | --- | --- | --- | --- | --- |
| cohort_resid | -0.162 |  |  |  |  |  |
| age_scaled | -0.045 | -0.238 |  |  |  |  |
| fr_scaled | -0.013 | -0.014 | 0.076 |  |  |  |
| swwt | 0.603 | 0.048 | -0.042 | 0.091 |  |  |
| cost_scaled | 0.018 | 0.012 | -0.035 | 0.097 | -0.022 |  |
| fall_fear | 0.384 | -0.010 | -0.010 | -0.041 | -0.045 | 0.010 |
